# Bayesian analysis of static light scattering data for globular proteins

**DOI:** 10.1371/journal.pone.0258429

**Published:** 2021-10-14

**Authors:** Fan Yin, Domarin Khago, Rachel W. Martin, Carter T. Butts

**Affiliations:** 1 Department of Statistics, University of California at Irvine, Irvine, CA, United States of America; 2 Structural Biophysics Laboratory, Center for Cancer Research, National Cancer Institute, Frederick, Maryland, United States of America; 3 Departments of Chemistry and Molecular Biology and Biochemistry, University of California at Irvine, Irvine, CA, United States of America; 4 Departments of Sociology, Statistics, Computer Science and EECS and Institute for Mathematical Behavioral Sciences, University of California at Irvine, Irvine, CA, United States of America; Marche Polytechnic University, ITALY

## Abstract

Static light scattering is a popular physical chemistry technique that enables calculation of physical attributes such as the radius of gyration and the second virial coefficient for a macromolecule (e.g., a polymer or a protein) in solution. The second virial coefficient is a physical quantity that characterizes the magnitude and sign of pairwise interactions between particles, and hence is related to aggregation propensity, a property of considerable scientific and practical interest. Estimating the second virial coefficient from experimental data is challenging due both to the degree of precision required and the complexity of the error structure involved. In contrast to conventional approaches based on heuristic ordinary least squares estimates, Bayesian inference for the second virial coefficient allows explicit modeling of error processes, incorporation of prior information, and the ability to directly test competing physical models. Here, we introduce a fully Bayesian model for static light scattering experiments on small-particle systems, with joint inference for concentration, index of refraction, oligomer size, and the second virial coefficient. We apply our proposed model to study the aggregation behavior of hen egg-white lysozyme and human *γ*S-crystallin using in-house experimental data. Based on these observations, we also perform a simulation study on the primary drivers of uncertainty in this family of experiments, showing in particular the potential for improved monitoring and control of concentration to aid inference.

## Introduction

For proteins in aqueous solution, measuring association states and propensities towards/away from aggregation is essential for understanding the formation and evolution of both native quaternary structure and deleterious aggregation, due to the fundamental roles of these properties in protein association [1–4]. Unfortunately, this is difficult, particularly in the highly relevant case of systems at low concentration at or near physiological pH. Current state-of-the-art approaches (e.g. small-angle X-ray scattering [5] and neutron scattering [6, 7]) require access to a beamline, which is typically located at a national laboratory or other remote facility. Sending samples to a beamline is expensive and must be scheduled far in advance, which limits the number of sample preparation conditions that can realistically be tested. A venerable but useful alternative is *static light scattering*, which can allow one to infer such critical quantities as aggregate size (and, in some cases, form factor) and local tendency towards or away from aggregation (as measured by osmotic pressure virial coefficients) [8, 9]. Unlike X-ray or neutron scattering, light scattering experiments can be performed with commercially available instruments within a typical lab setting [10–12], allowing for both greatly reduced cost and greatly enhanced flexibility.

A major barrier to the more widespread use of static light scattering for protein association assays is the lack of a modern, principled approach to data analysis. In the context of soluble proteins (and small oligomers or aggregates thereof), successful inference depends on both error reduction (via a combination of careful experimental procedure and data preparation) and leveraging of prior physical information. Standard approaches within the field, by contrast, are ad hoc and largely depend on graphical techniques developed in the 1940s-1950s [13]. These methods provide no principled estimates of uncertainty, and are unable to fully leverage the information content of the available data (e.g., exploiting the consistency between multiple different types of measurements involving the same quantities). A more modern approach to data analysis could greatly expand the reach of this approach, making it a viable alternative to small-angle X-ray and related measurements for protein research.

In this paper, we address this gap by introducing a systematic approach to the processing and analysis of data from static light scattering experiments on proteins and protein oligomers. This approach can be generalized to larger protein aggregates and/or other polymers, although we focus on the case of small to medium-sized globular proteins.

Our approach consists of two general elements. First, we employ a robust data cleaning and pre-processing scheme to find and remove experimental artifacts from the data (see Section 1 in S1 File for details). This scheme is intended to be largely automated, with minimal supervision from the analyst required to verify that the data have been properly processed. Having processed the raw observations, we then employ a hierarchical Bayesian model to correct for known sources of error and infer quantities of scientific interest. At the core of this model is a joint treatment of light scattering and refractive index data (the latter being required for analysis of light scattering experiments) in a way that allows all available information to be leveraged for inference.

The structure of this paper is organized as follows. The rest of this section offers a brief overview on the theory of light scattering and conventional approaches to data analysis. The section that follows describes the proposed Bayesian model in detail, which is applied to study the aggregates of two soluble, globular proteins: lysozyme and human *γ*S-crystallin, in the following two sections, respectively. Following a discussion of these results, we build on the findings of these empirical case studies with a series of simulation experiments to understand the impact of sample size and adjusting for measurement error in concentration measurements on inferential accuracy, the results of which provide critical insights for future experiments. We close with a general discussion and conclusion.

### Small particle scattering: Theoretical and experimental background

Static Light Scattering (SLS) provides information regarding (variously) mass, radius of gyration, or interaction propensity among particles in solution, by exploiting the way these properties affect the scattering of incident light [14–16]. Specifically, if a sample is illuminated by a beam of light at a fixed angle and wavelength, the intensity of light scattered at some angle *θ* relative to the angle of incidence is a function of the properties of the scatterer, allowing the latter to be inferred from the former. This intensity is usually referred to in terms of the *Rayleigh ratio*, *R*_*c*,*θ*_, an observable function of the intensity of the light detected at angle *θ* relative to the intensity of the incident beam [17]. In practice, the Rayleigh ratio depends upon the concentration of the solute (*c*), among other quantities; although its exact behavior is complex, for the specific case of small particles in dilute solution it can be approximated as [10, 13]

$$\begin{array}{c} R_{c,\theta }\approx K^{*}M_{w}P\left(r_{g},\theta \right)c-2K^{*}A_{2}M_{w}^{2}P{\left(r_{g},\theta \right)}^{2}c^{2}+O\left(c^{2}\right), \end{array}$$ (1)

or alternatively in its reciprocal form as [18]

$$\begin{array}{c} \frac{K^{*}c}{R_{c,\theta }}\approx \frac{1}{M_{w}P\left(r_{g},\theta \right)}+2A_{2}c+O\left(c^{2}\right) \end{array}$$ (2)

where *P*(*r*_*g*_, *θ*) is an angular dependence factor, *c* is the concentration of the solute, K* is a material constant, *M*_*w*_ is the *weight* average molecular weight of the scattering particle (defined Σ_*j*_*W*_*j*_*M*_*j*_, where *W*_*j*_ is the fraction of the total solute weight represented by each chemical species *j*, e.g. monomers, dimers, etc,. each with molecular weight *M*_*j*_, summed over all species present), and *A*_2_ is the second virial coefficient, a key physical constant of substantial scientific interest, governing the strength of pairwise interactions among particles. An intuitive explanation of (1) is that the scattering intensity can be approximated to first order by the mass and concentration of particles from which the beam can scatter (linear term), with a second-order effect arising from the the pairwise interactions among particles (quadratic term): particles that tend to cluster (*A*_2_ < 0) act “larger,” on average, generating a stronger signal, while particles that avoid each other (*A*_2_ > 0) produce fewer clusters and lower scattering intensity. Higher-order virial coefficients (e.g., *A*_3_) govern the contributions from higher-order interactions among greater numbers of particles; in dilute solution, such effects are small and exceedingly difficult to measure, and as such their contribution is generally discarded.

In addition to the effect of concentration and particle interaction, the Rayleigh ratio depends upon two other factors. The material constant K*,

$$K^{*}\equiv \frac{4\pi^{2}n_{0}^{2}{\left(dn/dc\right)}^{2}}{N_{A}\lambda^{4}}$$

depends on the intrinsic properties of the materials used for the experiments, and of the light source: the wavelength of the incident light λ, the refractive index of the solvent *n*_0_, the refractive index increment, i.e. *dn*/*dc* of the solute/solvent pair, the mathematical constant *π*, and Avogadro’s number, *N*_*A*_ = 6.022 × 10^23^ / mol. In general, the intensity of the scattered light also depends on an angular dependence factor *P*(*r*_*g*_, *θ*),

$$P{\left(r_{g},\theta \right)}^{-1}\approx 1+\frac{16\pi^{2}}{3\lambda^{2}}{\left\langle r_{g}^{2}\right\rangle }_{w}{\text{sin}}^{2}\left(\frac{\theta }{2}\right),$$

where ${\langle r_{g}^{2}\rangle }_{w}$ is weight average squared radius of gyration. For large scatterers (e.g., polymers) with radii comparable to the wavelength of the light source, *P*(*r*_*g*_, *θ*) can vary appreciably. In the case of small particles, however, where ${\langle r_{g}^{2}\rangle }_{w}\ll \lambda_{2}$, *P*(*r*_*g*_, *θ*)^−1^ ≈ 1 and angular effects can be ignored.

In this paper, we are specifically interested in the use of SLS to study aqueous solutions of non-aggregating globular proteins at low concentration (typically on the order of 10 mg/mL), under illumination by visible light (λ = 657 × 10^−7^ cm). In this regime, the second-order approximation of Eq (1) holds, and we may focus exclusively on pairwise interactions between particles without considering the higher-order interactions involving multiple particles that can occur in crowded solutions. Moreover, as these particle sizes are on the order of 10^−7^cm, angular dependence on scattering is below the detection limit of typical instruments, and we hence take *P*(*r*_*g*_, *θ*)^−1^ = 1 throughout. Without loss of generality, we work with the Rayleigh ratio measured at angle *θ* = 90° with respect to the incident beam. Because we work at a constant measurement angle (and the regime of interest is not angle-dependent), we henceforth simplify notation by dropping reference to *θ* and *P*(*r*_*g*_, *θ*) in the remainder of the paper except as noted otherwise.

#### Important sources of errors

Because the second virial coefficient (i.e. *A*_2_) represents a very small deviation in local effective particle density (relative to uniform mixing), it is challenging to estimate with high precision. Eqs (1) and (2) shows that estimating *A*_2_ requires knowledge of the concentration *c*, refractive index increment *dn*/*dc*, and Rayleigh ratio *R*_*c*_, all of which are prone to measurement errors of different types and magnitudes. To obtain an accurate point estimate and evaluation of the uncertainty of *A*_2_, accounting for these measurement errors is of substantial importance. Modeling these errors requires a careful consideration of the experimental procedure used to produce the associated measurements; we discuss this in more detail below.

#### Units of measurement

The units of the physical quantities involved in this analysis are listed (or can be derived using those listed) in Table 1. Unless otherwise specified, the units of physical quantities remain the same as listed in Table 1 for the rest of this paper.

**Table 1:** Units of measurement. The physical quantity with unit of measurement 1 is unitless.

Physical quantity	Unit of measurement	Description
*R*_*c*,*θ*_	1/cm	Rayleigh ratio
*n*_0_	1	Refractive index of solvent
*c*	g/mL	Concentration of of the solute
*dn*/*dc*	mL/g	Refractive index increment
λ	cm	Wavelength of incident light
*A*_2_	mL mol/g^2^	Second virial coefficient
*N*_*A*_	6.022 × 10^23^/mol	Avogadro’s number
*M*_*w*_	g/mol	Weight average molecular weight

### Standard approaches to data analysis

Conventionally, with refractive index increment (*dn*/*dc*) and weight average molecular weight *M*_*w*_ assumed to be known in advance (or assumed to be accurately measured using other means), and the concentration being measured accurately, SLS data have been analyzed based on the “Zimm plot,” a two-stage regression proxy method developed by physical chemists based on Eq (2). Despite its popularity, which is primarily due to simplicity and ease of use, the Zimm plot cannot provide valid uncertainty estimates and can be numerically unstable. It can also be sensitive to measurement error, particularly with respect to concentrations (which can be difficult to calibrate precisely); more subtly, concentration enters into estimation of both *dn*/*dc* and *A*_2_, leading to complex correlations among errors. Some of these limitations can be mitigated by more principled statistical methods, such as the joint bootstrapped regression combining SLS and refractive index measurements introduced in [19]. Although this scheme provides a basis for obtaining confidence intervals for *A*_2_, and incorporates the interdependence of *dn*/*dc* and *A*_2_ estimation, it depends on the assumption of monodispersity (i.e., all scattering particles contain approximately the same number of monomers), and does not offer avenues for incorporation of prior information regarding either estimands or measurement error. Given, on the one hand, the need to leverage as much information as possible to facilitate precise measurements of *A*_2_ from limited experimental data, and, on the other, the availability of substantial physical knowledge regarding model parameters, this last is a consequential limitation.

The need for a combined treatment of *dn*/*dc*, *A*_2_, and particle size distribution is key to the limitations of heuristic strategies (such as the Zimm plot). In general, the common assumptions that the refractive index increment (*dn*/*dc*) and weight average molecular weight *M*_*w*_ will be known *ex ante* with high precision are not realistic. The refractive index increment, *dn*/*dc*, is often approximated by a pre-specified constant (a “standard” value based on a reference protein such as bovine serum albumin, or some other conventional “average”), or in more sophisticated cases a theoretical calculation based on refractivity of individual amino acids in solution [20]; both have been shown to have limited accuracy, especially for proteins such as the crystallins of the eye lens that are selected for high refractive index [21]. In practice, accurate assessment of *dn*/*dc* hence requires that it be independently measured with a separate instrument, leading to its own source of measurement error.

The situation for *M*_*w*_ is similar: while some proteins can be safely assumed to be monomeric in dilute solution, SLS is frequently used specifically to investigate proteins that are prone to aggregation and/or the formation of complex oligmeric states. In such cases, the size of the scattering particles is generally unknown, and even monodispersity may be difficult to guarantee (i.e., one may have a mix of oligomeric states). While (as we show) *M*_*w*_ can itself be estimated from SLS data, errors in this estimate are obviously intertwined with errors in the estimation of *A*_2_ (which depends on it). As noted above, estimation for all of these quantities depends upon solute concentration, which itself is imperfectly known. Concentration can be estimated from refractive index data, but this depends on knowledge of *dn*/*dc* which, as noted, is itself uncertain. We thus face a situation in which we have several linked unknowns, which must be resolved by leveraging multiple types of measurements simultaneously. Procedures that ignore such uncertainties by simply fitting to nominal values of concentration, *dn*/*dc*, or *M*_*w*_ may lead to seriously biased estimates and misleading uncertainty estimates [22, 23].

To incorporate a priori scientific knowledge into the analysis, as well as to effectively account for interacting measurement errors in a principled and unified way, we here propose a fully Bayesian model for analyzing the SLS data based on the idea in *errors-in-variable* (EIV) modeling framework [24, 25]. This model is specified in detail below.

## Bayesian inference for light scattering data

Given processed data from an SLS experiment, we develop a full Bayesian model for statistical inference for *A*_2_, *dn*/*dc*, and related quantities. A Bayesian modeling framework is particularly suitable for this problem, as it allows us to efficiently express the complex dependence among the physical quantities in the system, and to incorporate physical information regarding both parameters and the measurement process. Bayesian model construction also allows us to naturally emulate the logical structure of the experiment itself, with a clear representation of the flow of information from the different measurement processes and priors into the unknown model parameters. Finally, Bayesian answers for quantities such as posterior uncertainty in *A*_2_ values are especially useful given that even high-quality experiments typically estimate *A*_2_ with limited precision, and the range of *a posteriori* plausible *A*_2_ values is important for tasks such as comparison with simulation studies (e.g., [19]). Here, we proceed by first describing the structure of the model, followed by prior specification and implementation. The succeeding sections demonstrate applications to protein data, and provide a simulation experiment probing sensitivity to sample size and data quality.

### Model structure

**Fig 1:**
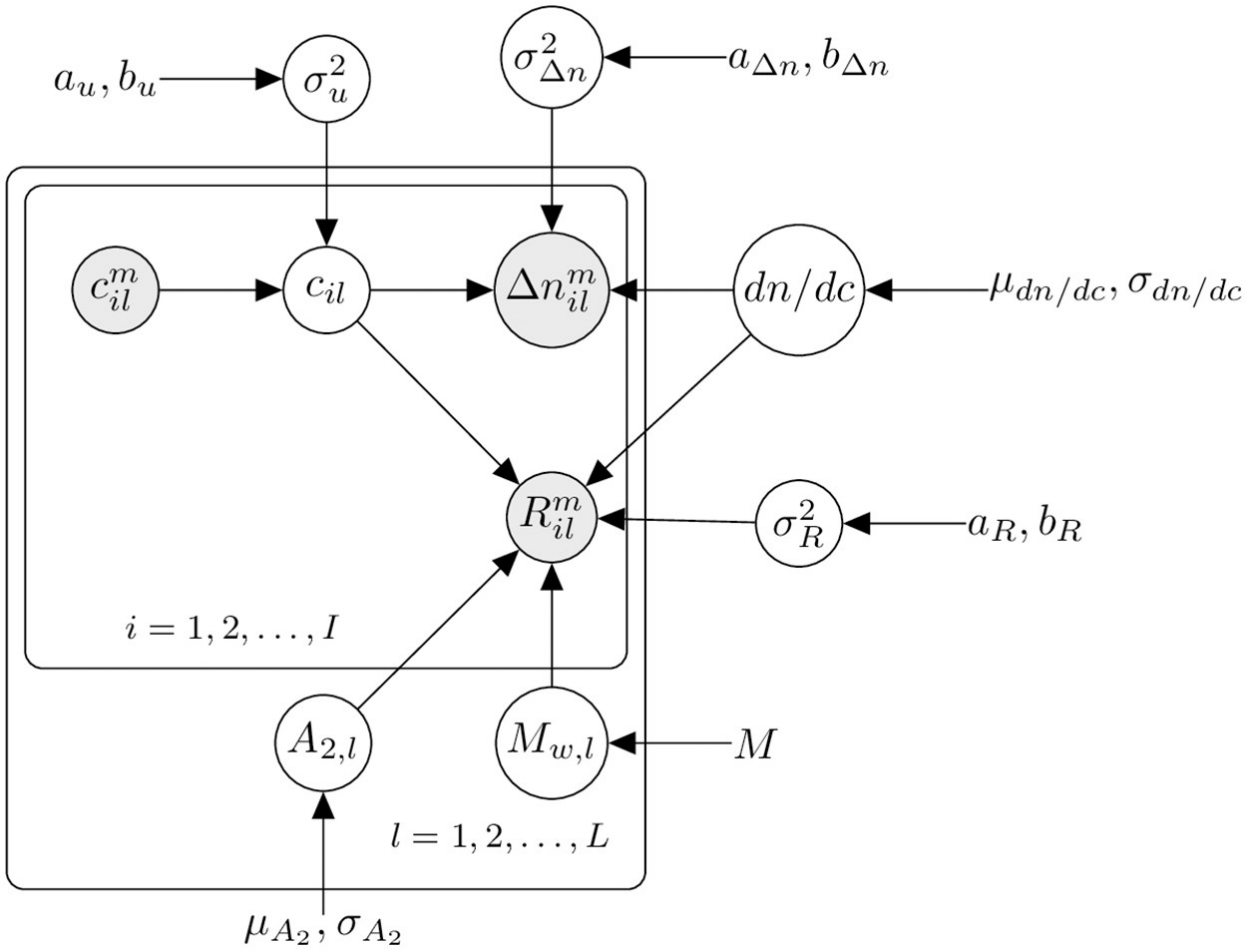
Structure for the Bayesian SLS model. Outer plate reflects distinct experimental conditions (e.g., variation in solution conditions), while inner plate reflects measurements at distinct concentrations. Measured quantities shown as shaded circles, with latent variable as unshaded circles; hyperparameters are shown as uncircled quantities.

We assume data in the form of measurements taken under *L* distinct solution conditions (e.g., ionic concentration, pH, etc.), at *I* distinct concentrations. For every condition *l* and concentration *i*, we observe a concentration measurement $c_{il}^{m}$, a refractive index measurement (i.e., measured refractive index minus solvent refractive index) Δ*n*_*il*_, and light scattering measurement *R*_*il*_. The plate diagram of Fig 1 shows the structure of the proposed model, which we explain in this section.

We begin by incorporating known physical constraints. First, we observe that the change in refractive index of the solution (versus solvent), Δ*n*_*il*_, is proportional to the sample concentration, i.e.

$$\begin{array}{c} \Delta n_{il}=n_{il}-n_{0l}=c_{il}\times dn/dc \end{array}$$ (3)

where *c*_*il*_ is the true concentration (g/mL) corresponding to measured concentration $c_{il}^{m}$, and *dn*/*dc* is the refractive index increment (i.e., the change in refractive index per concentration increment). We take *dn*/*dc* to be constant over the conditions of interest (as is generally the case). The formula for Rayleigh scattering *R*_*il*_ (1/cm) follows that of (1),

$$\begin{array}{c} R_{il}\approx K^{*}M_{w,l}c_{il}\left(1-2A_{2,l}M_{w,l}c_{il}\right) \end{array}$$ (4)

where *A*_2,*l*_ is the second virial coefficient (mol mL/g^2^) and *M*_*w*,*l*_ (Da; g/mol) is the weight average molecular weight under experimental condition indexed *l*. (For simplicity of notation, we shall omit the units when specifying the models.) The material constant K*, and its important parameters are described in the Introduction. In our experiments, we take the wavelength of the incident light to be fixed at the value reported in the instrument manual (λ = 657 × 10^−7^ cm), and we treat *n*_0_ as fixed because it can be accurately determined by repeated measurements using a high-precision refractometer. Therefore, the only random element of K* is *dn*/*dc*.

We model the observed readings of the light scattering (LS) and refractive index (RI) detectors, $R_{il}^{m}$ and $\Delta n_{il}^{m}$, as independent Gaussian and truncated Gaussian random variables centered at the respective theoretical values given by (4) and (3),

$$\begin{array}{c} R_{il}^{m}∼N\left(R_{il},\sigma_{R}^{2}\right) \end{array}$$ (5)

where $\sigma_{R}^{2}$ is the inverse precision of the scattering measurement, and

$$\begin{array}{c} \Delta n_{il}^{m}∼{TN}_{\left(0,\infty \right)}\left(\Delta n_{il},\sigma_{\Delta n}^{2}\right) \end{array}$$ (6)

with $\sigma_{\Delta n}^{2}$ likewise being the inverse precision of the refractive index measurement. We take these precisions to be constant across measurements. Restricting $\Delta n_{il}^{m}$ to be non-negative reflects physical constraints, although in practice this will have little impact except near the dection limit.

The measured concentrations $c_{il}^{m}$ are obtained via UV absorption spectroscopy, a high-precision technique. However, the true concentration *c*_*il*_ may still depart from the measured concentration due to the presence of filters and effects from liquid handling (e.g., adhesion of protein to surfaces, effects from transferring the prepared samples from the test tube to the instrument, etc.) that arise after the measurement is made. To account for these effects, we consider a multiplicative, Berkson-type measurement error model [26],

$$\begin{array}{c} c_{il}=c_{il}^{m}u_{il}, \end{array}$$ (7)

where *u*_*il*_ is independent of $c_{il}^{m}$ and has a lognormal distribution

$$\begin{array}{c} u_{il}\overset{iid}{∼}LN\left(0,\sigma_{u}^{2}\right), \end{array}$$ (8)

with $\sigma_{u}^{2}$ reflecting the log-variance of concentration discrepancies.

Combining (5), (6), (7) and (8) together, we note that our model can be viewed as a multivariate response quadratic regression ($R_{il}^{m}$, $\Delta n_{il}^{m}$) with multiplicative measurement error in the explanatory variable *c*_*il*_. It is also worth mentioning that the aggregation status of the protein of interest is unknown (and is often the subject of intense interest) in some experimental settings, and our proposed framework is flexible enough to allow for statistical inference on *M*_*w*_; we discuss this in our sample applications.

### Prior specification

We assign Gaussian priors to *dn*/*dc*,

$$\begin{array}{c} dn/dc∼N(\mu_{dn/dc},\sigma_{dn/dc}^{2}), \end{array}$$ (9)

with its location and scale being determined using literature values. To facilitate statistical inference with a parsimonious model, we assume that *dn*/*dc* for a specific type of protein is unchanged across different experimental conditions, which is plausible under the conditions covered in our experiments in Table 2. A hierarchical structure on *dn*/*dc* can be adopted when the NaCl concentrations and pH values have larger spans. We then assign inverse-gamma priors to variance parameters,

$$\begin{array}{cc} \sigma_{u}^{2} & ∼IG(a_{u},b_{u})\\ \sigma_{R}^{2} & ∼IG(a_{R},b_{R})\\ \sigma_{\Delta n}^{2} & ∼IG(a_{\Delta n},b_{\Delta n}) \end{array}$$

with the shape and rate parameters chosen based on precisions reported by the instrument manufacturers and concentrations based on the strength of prior belief in said reports (this type of intuitive specification being facilitated by use of an exponential family). The prior for the second virial coefficient, *A*_2_, is set to be a Gaussian distribution,

$$A_{2,l}\overset{iid}{∼}N\left(\mu_{A_{2}},\sigma_{A_{2}}^{2}\right),$$

where we choose $\mu_{A_{2}}=0$ and $\sigma_{A_{2}}=1$ (in the units of Table 1) to reflect that *A*_2_ can be either positive or negative, and to provide a prior that is fairly flat over the physically plausible range (magnitudes less than ≈ 10^−2^mL mol/g^2^) without imposing hard upper or lower limits.

### Connections with other models

The proposed model assumes a lognormal-based multiplicative Berkson-type measurement error in the concentrations, which can be viewed as “explanatory variables” from a regression modeling standpoint. To the best of the authors’ knowledge, the model structure of Fig 1 is novel—it is different from well-established statistical procedures [25, 27] that focus on classical measurement error, and it is also different from the literature on additive Berkson-type measurement error [28, 29] and prior work on bounded multiplicative Berkson-type measurement errors [30].

We note that the proposed model is well-posed from a Bayesian perspective in the sense that the posterior distribution of $\sigma_{u}^{2}$ can be estimated as long as the prior for $\sigma_{u}^{2}$ is a legitimate probability distribution [31, 32]. We consider the issue of posterior precision given sample size and data quality in the simulation study that follows our empirical case studies.

### Implementation

All computations in this paper were performed in R (version 4.0.1) [33] on a computing server (256GB RAM, with 8 AMD Opteron 6276 processors, operating at 2.3 GHz, with 8 processing cores in each). We used the library R2jags (version 0.6.1) [34] and the JAGS sampler software (version 4.3.0) [35] for conducting Markov Chain Monte Carlo (MCMC) sampling in both case studies and simulation experiments. We note that the proposed model can be implemented using other commonly-used, open-access tools such as WinBUGS [36] and Stan [37].

## Application to aggregation propensity assessment in lysozyme

In this section, we apply the Bayesian model to analyze SLS data collected from experiments on lysozyme, an antimicrobial enzyme produced by animals that forms part of the innate immune system. Lysozyme can be either aggregation resistant or aggregation prone under particular conditions, and is a common model system for protein aggregation studies [1, 38, 39]; determining the solution conditions under which *A*_2_ switches from positive (repulsive interactions) to negative (attractive interactions) is a point of particular interest. Here, we examine this question in the context of experiments that vary both pH (altering protonation states, and hence both protein fold and surface charge distribution) and ionic concentration (affecting charge screening, and the stability of salt bridges).

### Experimental conditions and data collection

Lyophilized hen egg white lysozyme was purchased from MP Biomedicals (Solon, OH), and the lysozyme was first weighed and then dissolved in 10 mM sodium phosphate (pH 4.7 and 6.9) containing 0.05% sodium azide and sodium chloride (i.e., NaCl) concentrations at 50, 75, 100, 125, 150, 200, 250, and 300 mM for a target protein concentration of 50mg/mL. This stock solution was then diluted sequentially to produce solutions with nominal lysozyme concentrations of 2.5, 5, 7.5, 10, 12.5, 15, 17.5, 20, 25, 30, 35, 40, 45, and 50 mg/mL, a total of 14 concentration levels. The concentrations were measured by UV absorbance spectroscopy using a Cary 7000 spectrophotometer (Agilent Technologies, Santa Clara, CA). A molar absorptivity coefficient of *ϵ* = 2.64 mL mM^−1^ cm^−1^ at 280nm was used. Refractive index increments were measured using a batch-mode technique with an Optilab rEX refractive index detector (Wyatt Technology, Santa Barbara, CA). Light scattering measurements were performed using a Wyatt Instruments Dawn HELEOS multi angle light scattering (MALS) instrument. (Wyatt Technology, Santa Barbara, CA).

Table 2 gives a list of experimental conditions. The value in each cell of Table 2 indicates the number of experimental runs under each respective condition. There are two pH levels (4.7, 6.9) and eight NaCl concentration levels (50, 75, 100, 125, 150, 200, 250, and 300 mM) by the original design; however, the data for the experiments under conditions (pH = 4.7, NaCl:200mM and 250mM) could not be obtained. As a result, the SLS experiments for lysozyme have a total of 14 experimental conditions (varying pH and salt concentration), each of which has one run with 14 lysozyme concentration levels.

**Table 2:** Experimental conditions for lysozyme. Values in each cell indicate the number of experimental runs (solution *n*_0_ in parentheses) under respective condition.

NaCl (mM)	50	75	100	125	150	200	250	300
pH = 4.7	1 (1.3272)	1 (1.3272)	1 (1.3285)	1 (1.3279)	1 (1.3276)	0	0	1 (1.3296)
pH = 6.9	1 (1.3291)	1 (1.3295)	1 (1.3273)	1 (1.3289)	1 (1.3309)	1 (1.3312)	1 (1.3331)	1 (1.3310)

### Data preparation

**Fig 2:**
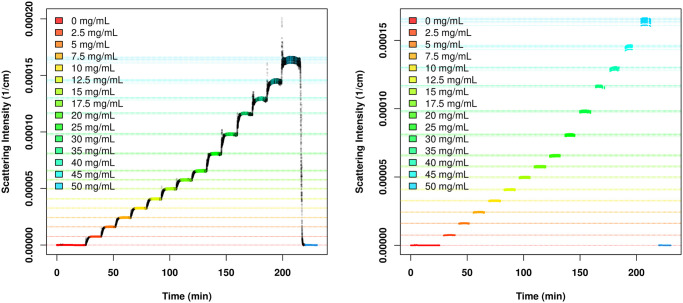
(Left) Light scattering data for lysozyme in 10 mM phosphate, 100 mM sodium chloride, 0.05% sodium azide at pH 6.9. Scattering intensity is recorded over the time of the experiment. Each color represents a particular concentration of lysozyme being injected into the MALS instrument (by design concentration), with the first and last being buffer for baseline correction; each line reflects a different detector. Black areas indicate artifacts introduced by the sample injection (see supplement for processing details). (Right) Post-cleaning scatting intensity measurements; horizontal lines indicate trimmed median estimates used for subsequent analysis.

We remove the experimental artifacts, highlighted in black in Fig 2 (left panel), using the automatic data pre-processing algorithm detailed in Section 1 in S1 File. The pre-processed data are shown in the right-hand panel of Fig 2. and the median scattering intensity at 90° was used as the Rayleigh ratio measurement for each concentration. This procedure could also be generalized to multiple detectors if desired.

We note that the RI detector produced unphysical values for relatively high concentrations (due to exceedance of its upper detection limit), and we thus only included refractive index measurements from nominal lysozyme concentrations no greater than 20 mg/mL. Within this range, all LS measurements are included in the analysis because the LS detector gives physically valid measurements across the entire concentration range after removing the artifacts. Table 3 shows which LS and RI measurements are included in the analysis for each experimental run—within each experimental run, only the eight lowest concentration levels give valid refractive index measurements, whereas all the concentration levels provide valid Rayleigh ratio measurements.

**Table 3:** LS and RI measurements under different nominal concentration levels within an experiment replicate. “Y” indicates the corresponding signal is included in the analysis, otherwise “N”.

level (i=)	1	2	3	4	5	6	7	8	9	10	11	12	13	14
concentration (mg/mL)	2.5	5	7.5	10	12.5	15	17.5	20	25	30	35	40	45	50
RI	Y	Y	Y	Y	Y	Y	Y	Y	N	N	N	N	N	N
LS	Y	Y	Y	Y	Y	Y	Y	Y	Y	Y	Y	Y	Y	Y

### Model specification

Current understanding of the aggregation states of lysozyme in solution under these experimental conditions suggests that dimers are likely to be the dominant oligomeric species under the conditions studied here [40, 41]. Thus, we propose the following competing models for the aggregation state (expressed via the mass-averaged molecular weight, *M*_*w*_):

-
$M_{1}:M_{w,l}=M$

-
$M_{2}:M_{w,l}=2M$

-
$M_{3}:M_{w,l}=\left(k_{l}+1\right)M,\;k_{l}\overset{iid}{∼}Bern\left(0.5\right)$

-
$M_{4}:M_{w,l}\overset{iid}{∼}U\left(M,2M\right)$

where *M* = 14307 g/mol is the molar mass of a lysozyme monomer, *Bern* is the Bernoulli distribution, and *U* is the continuous uniform disribution. *The first three models assume monodispersity—that is, the scattering particles within each experimental condition are exclusively monomers or dimers—with $M_{1}$ and $M_{2}$ additionally assuming homogeneity across conditions and $M_{3}$ allowing aggregation state to vary by condition*. As it is of substantial interest to explore whether this assumption of monodispersity is supported by experimental data or not, the fourth model relaxes the monodispersity assumption by allowing the weight average molecular weight to take continuous values between the weight of monomers and dimers (with this value being permitted to vary by condition). A data-driven answer to this scientific question can be facilitated by model selection techniques; specifically, we employ the Deviance Information Criterion (DIC) [42] for this purpose, which can be automatically evaluated by the R function bugs in the package R2WinBUGS [43]. The DIC is a generalization of the well-known Akaike’s Information Criterion [44], both of which are model selection criteria that attempt to assess the extent to which a model will generalize well to new data (in the sense of the log likelihood of a hypothetical replicated data set) by penalizing the observed model fit by a term related to model complexity (its effective degrees of freedom, and hence tendency to over-fit). Competing models with lower DIC are expected to have better generalization performance (in this sense), and are preferred.

To conduct posterior inference, we need to specify hyperparameter values for the prior distribution. We do so as follows:

-
$dn/dc∼{TN}_{\left(0,\infty \right)}\left(\mu_{dn/dc}=0.1970,\sigma_{dn/dc}^{2}=0.005^{2}\right)$ , reflecting the prior knowledge that the mean of the refractive index increment of lysozyme is about 0.1970 [45] and the refractive index increment of globular proteins is non-negative and has a range of about ±0.03 [21].
-
$\sigma_{R}^{2}∼IG\left(a_{R}=1,b_{R}={\left(10^{-5}\right)}^{2}\right)$ and $\sigma_{\Delta n}^{2}∼IG\left(a_{\Delta n}=1,b_{\Delta n}={\left(10^{-4}\right)}^{2}\right)$, reflecting the prior knowledge that the precision level of LS and RI measurements should have order of magnitude 10^−5^ and 10^−4^, respectively, while there is also considerable probability that the precision can go beyond or below the nominal level.
-
$\sigma_{u}^{2}∼IG\left(a_{u}=1,b_{u}={\left(\text{log}\left(1+0.05\right)/1.96\right)}^{2}\right)$ , reflecting the belief (based on the experimenters’ experience with similar sample preparation protocols) that the true concentration should be between 95% and 105% of the measured value with fairly large probability.

### Results

For each candidate model, we run 5 independent Markov Chain Monte Carlo (MCMC) chains with random starting values and conservative settings (300000 total MCMC iterations, burn-in 200000, storing every 250-th iteration of the last 100000 draws as posterior samples). Visual inspection of the trace plots and the Brooks-Gelman-Rubin statistic [46, 47] shows that the chains mix well (For a general introduction to Bayesian MCMC procedures, see e.g. [48].) These independent chains are run in parallel, with each chain taking approximately 10.50, 13.56, 12.25, and 13.27 minutes for $M_{1}$, $M_{2}$, $M_{3}$ and $M_{4}$, respectively.

Table 4 presents the DIC values for the competing models, indicating that $M_{1}$ and $M_{3}$ fit the data equally well and are substantially better than other competing models. Further investigations on the posterior samples under $M_{3}$ show that the *k*_*l*_, *l* = 1, …, 14 all converge to 0 (i.e., *M*_*w*,*l*_ = (*k*_*l*_ + 1)*M* = (0 + 1)*M* = *M*). These results favor the assumption that lysozyme is in the monomeric form under these experimental conditions, and hence we select $M_{1}$ as the model for subsequent inferential analysis on *A*_2_.

**Table 4:** DIC values for candidate models for lysozyme solution. Optimal model with lowest DIC value is highlighted in bold.

	$M_{1}$	$M_{2}$	$M_{3}$	$M_{4}$
DIC	**-5447.9**	-4462.1	**-5449.3**	-5244.8

Table 5 presents several summary statistics for the posterior samples of *A*_2,*l*_, *l* = 1, …, 14. Under each fixed pH, we observe an overall downward trend of *A*_2_ values, which is in line with the theory that interactions between monomers become less repulsive as the ionic strength in the solution becomes stronger (i.e., higher NaCl concentrations). Under fixed NaCl concentration, smaller *A*_2_ is associated with a higher pH, consistent with the observation that protein solubility is decreased when approaching the isoelectric point (which is strongly basic for lysozyme). Interestingly, this downward pattern is slightly violated when the NaCl concentrations are at 200mM and 250mM (perhaps reflecting a change of conformational state), providing a target for protein structure modeling studies. As shown in the rightmost column of Table 5, we have high posterior certainty that the pairwise interaction between lysozyme monomers is repulsive (i.e., *P*(*A*_2_ > 0|⋅) ≈ 1) under low NaCl concentrations (pH = 4.7, NaCl: 50, 75, 100, 125 mM; pH = 6.9, NaCl: 50, 75 mM), and fairly high certainty that the pairwise interaction between lysozyme monomers is attractive (i.e., *P*(*A*_2_ > 0|⋅) < 0.01) under high NaCl concentrations (pH = 4.7, NaCl: 300mM; pH = 6.9, NaCl: 125, 150, 200, 250, 300 mM). These findings confirm the previous experimental observations that high salt conditions promote attractive interaction and hence e.g., crystallization [1].

**Table 5:** Posterior mean, standard deviation (SD), 2.5% and 97.5% quantile of *A*_2_ × 10^5^ under various pH and NaCl strength conditions for lysozyme. The units of *A*_2_ are mL mol/g^2^. The probability of *A*_2_ being positive is also presented. The numbers in parentheses are the results under the “no adjustment” model.

*l* =	pH	NaCl (mM)	Mean	SD	2.5% quantile	97.5% quantile	*P*(*A*_2_ > 0|⋅)
1	4.7	50	35.521 (36.450)	1.3 (2.1)	32.859 (32.693)	37.699 (40.891)	1.000 (1.000)
2	4.7	75	25.803 (26.512)	1.4 (1.8)	22.817 (23.089)	28.237 (29.958)	1.000 (1.000)
3	4.7	100	23.829 (24.431)	1.4 (1.9)	20.983 (20.716)	26.286 (28.052)	1.000 (1.000)
4	4.7	125	9.755 (9.708)	2.3 (2.0)	4.894 (5.926)	14.053 (13.628)	1.000 (1.000)
5	4.7	150	-2.681 (4.250)	3.4 (2.1)	-9.610 (-0.113)	3.479 (7.788)	**0.217**(**0.973**)
6	4.7	300	-7.654 (-6.199)	3.2 (2.4)	-14.163 (-11.333)	-1.979 (-1.809)	0.005 (0.000)
7	6.9	50	20.312 (20.713)	1.7 (2.1)	17.281 (16.541)	23.679 (24.699)	1.000 (1.000)
8	6.9	75	13.308 (14.295)	1.7 (1.6)	9.898 (11.341)	16.435 (17.081)	1.000 (1.000)
9	6.9	100	-0.630 (1.688)	2.8 (1.9)	-6.531 (-2.000)	4.607 (5.268)	0.427 (0.835)
10	6.9	125	-11.225 (-5.739)	3.6 (2.4)	-18.011 (-10.845)	-4.490 (-1.281)	0.003 (0.007)
11	6.9	150	-19.510 (-16.836)	3.8 (2.6)	-26.754 (-22.297)	-12.304 (-11.917)	0.000 (0.000)
12	6.9	200	-8.681 (-4.811)	3.5 (2.0)	-15.642 (-9.082)	-2.179 (-0.722)	0.003 (0.007)
13	6.9	250	-7.888 (5.490)	3.5 (1.3)	-15.181 (3.083)	-1.318 (8.170)	**0.007**(**1.000**)
14	6.9	300	-32.855 (-22.884)	4.4 (3.0)	-41.955 (-28.556)	-25.435 (-17.275)	0.000 (0.000)

**Fig 3:**
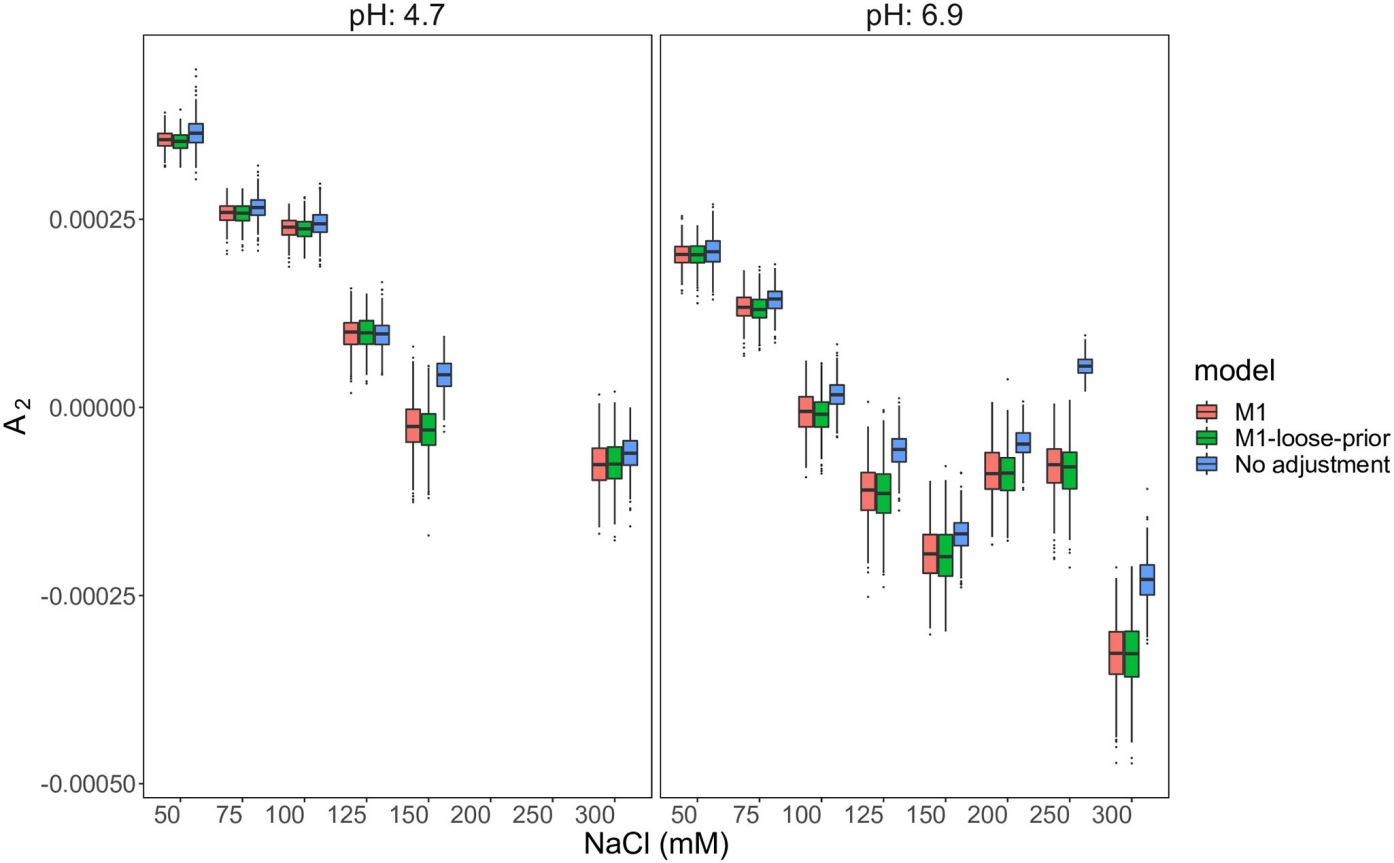
Boxplots of posterior samples (lysozyme): The second virial coefficients for lysozyme (*A*_2_) estimated from $M_{1}$ under different priors (red and green), and a model without concentration error adjustment (blue). Failing to account for error in concentrations leads to highly discrepant results, as well as underestimation of uncertainty in *A*_2_ values.

**Fig 4:**
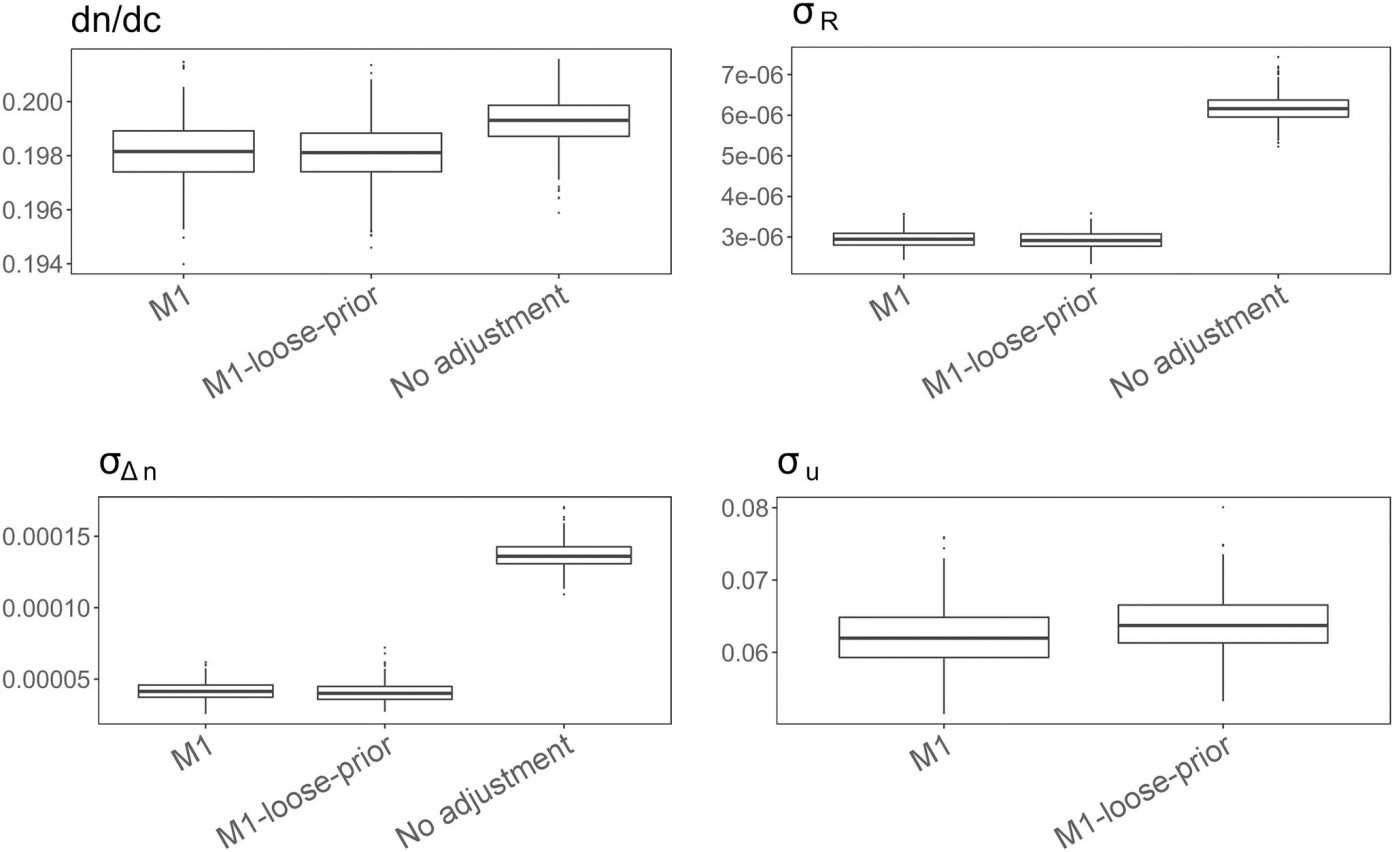
Boxplots of posterior samples (lysozyme): *dn*/*dc*, *σ*_*R*_, *σ*_Δ*n*_ and *σ*_*u*_ under $M_{1}$ and a model without concentration error adjustment. Assuming perfectly measured concentrations leads to overestimation of *dn*/*dc* and inflated estimates of instrument error.

We perform a sensitivity analysis with a much looser prior on $\sigma_{u}^{2}$ (i.e., *b*_*u*_ = (log(1 + 0.25)/1.96)^2^) to examine the robustness of our results. Figs 3 and 4 show the posterior samples of second virial coefficients (*A*_2_), *dn*/*dc*, *σ*_*R*_, *σ*_Δ*n*_ and *σ*_*u*_. These figures show that our results are in general not sensitive to loose-yet-meaningful priors on $\sigma_{u}^{2}$ (fairly large probability of true concentration falling between 75% and 125% of the measured concentration). As expected, we note that the posterior samples of *σ*_*u*_ are slightly larger under looser priors.

As a point of comparison, we also run a model without adjusting for measurement errors in concentrations, in which we treat the measured concentration as the true concentration. Table 5 shows that the model without measurement error adjustment results in very different point and interval estimates of *A*_2_, and gives almost opposite qualitative results for the changes in *A*_2_ (pH = 4.7, NaCl: 150mM; pH = 6.9, NaCl: 250 mM). As shown in Fig 4, such a model clearly forces the here unaccounted-for errors in concentration measurements to be propagated into other sources: *σ*_Δ*n*_ under the “no adjustment” model is estimated to be almost three times that of $M_{1}$, and *σ*_*R*_ under the “no adjustment” model is estimated to be almost two times that of $M_{1}$. These effects emphasize the need to account for concentration errors during analysis. We further illustrate the importance of adjusting for measurement errors via a simulation study in below.

We conduct posterior predictive checks [49] to examine whether posterior predictive samples of LS and RI readings can cover the measured values reasonably enough to be scientifically plausible (see Section 2 in S1 File for more details).

## Application to aggregation propensity assessment in human *γ*S-crystallin

In this section, we study the aggregation status of human *γ*S-crystallin (H*γ*S), a major structural component of the human eye lens. H*γ*S is noteworthy for its ability to remain in solution at the extremely high concentrations necessary to give the lens its refractive power, while resisting aggregation; indeed, as the lens contains no mechanisms to either remove or replace aggregated H*γ*S, it must remain in solution for one’s entire life [50–52]. Crystallin aggregation leads to cataract, the leading cause of blindness worldwide [53], and is hence of considerable scientific importance. The transient oligomerization states of H*γ*S along the path to aggregation are poorly understood beyond dimers [54, 55], and precise measurements of its *A*_2_ values under different solution conditions are so far lacking, making it a natural target for investigation using SLS.

### Experimental condition and data preparation

DNA encoding the sequence of human *γ*S-crystallin (UniProt ID: CRYGS_HUMAN) [56], codon-optimized for expression in *E. coli*, was purchased from Blue Heron (Bothell, WA). This gene was cloned into a pET28a(+) plasmid (Novagen, Darmstadt, Germany) containing an N-terminal 6× His tag and a TEV cleavage sequence (ENLFQG), which leaves a glycine in place of the initiator methionine. The protein was overexpressed in a Rosetta *E. coli* cell line (DE3) using autoinduction as described by [57]. Cell pellets were collected via centrifugation at 4,000 rpm for 30 minutes, resuspended, lysed, and spun again at 14,000 rpm for 60 minutes. Finally, the protein was purified via nickel affinity chromatography, digested with TEV protease (produced in-house), and the His-tag removed using a nickel affinity chromatography step. Three experiments were conducted under the same solution condition (pH = 6.9, NaCl = 100mM; this is similar to the environment of the human lens); the biophysical measurements were carried out in the same way as for lysozyme.

Table 6 shows the availability of RI and LS measurements for H*γ*S under different nominal concentration levels, which is similar to that of lysozyme—note that RI readings are not available for conditions with nominal concentrations > 20 mg/mL, due to limitations of the refractometer for proteins of particularly high refractive index.

**Table 6:** LS and RI measurements for H*γ*S under different nominal concentration levels within an experiment replicate under our experiment condition (pH = 6.9, NaCl concentration:100mM). “Y” indicates the corresponding signal is included in the analysis, otherwise “N”.

level (i=)	1	2	3	4	5	6	7	8	9	10	11	12	13	14
concentration (mg/mL)	0.5	1	2	3	4	5	7.5	10	12.5	15	17.5	20	25	30
RI	Y	Y	Y	Y	Y	Y	Y	Y	Y	Y	Y	Y	N	N
LS	Y	Y	Y	Y	Y	Y	Y	Y	Y	Y	Y	Y	Y	Y

The raw experimental data were cleaned before analysis, using the automatic procedure detailed in Section 1 in S1 File.

### Model specification

Although H*γ*S is generally monomeric in its functional state, it exists under very crowded conditions in the eye lens, where it avoids persistent aggregation despite having mildly attractive intermolecular interactions [58]. The current understanding of the *transient* oligomerization states of *γ*S-crystallin is limited; possibilities include both polydispersity and monodispersity with large, dynamically exchanging structures (scattering units). With this in mind we consider the following candidate models for *M*_*w*_ (here we omit the index *l* as we only have one pH and salt condition, which was chosen to mimic the physiological situation):

-
$M_{x}:M_{w}=xM$ , *x* = 1, 2, …, 20
-
$M_{21}:M_{w}∼U\left(M,20M\right)$

-
$M_{22}:M_{w}∼N\left(\mu_{M_{w}},\sigma_{M_{w}}^{2}\right)$ , where $\mu_{M_{w}}∼U\left(M,20M\right)$, $\sigma_{M_{w}}^{2}∼IG\left(1,{\left(\frac{M}{3}\right)}^{2}\right)$

where *M* = 20959.80 g/mol. Models $M_{x},\;\;x=1,2,\ldots ,20$ assume monodispersity (with particle sizes ranging from 1 to 20 monomers), while $M_{21}$ and $M_{22}$ allow the co-existence of different aggregation states. In model $M_{22}$, the prior for $\sigma_{M_{w}}^{2}$ is chosen to ensure that possible aggregates are close to the center. Other hyperparameters for the H*γ*S models were chosen as per the lysozyme analysis (i.e., the same values were employed).

### Results

**Fig 5:**
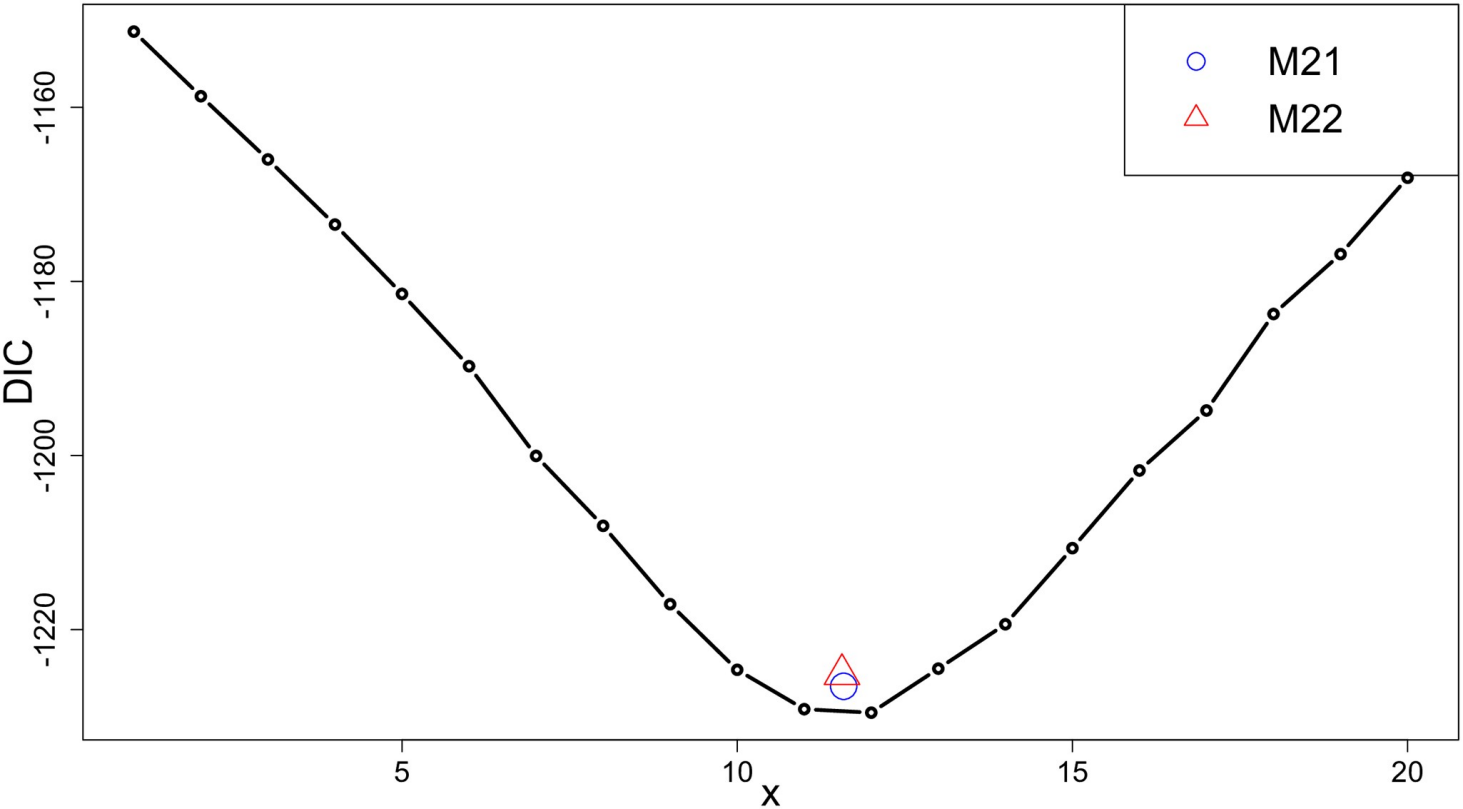
DIC values for $M_{x},\;x=1,2,\ldots ,20$, $M_{21}$ (blue circle) and $M_{22}$ (red triangle) for H*γ*S. The x-axis values of points associated with $M_{21}$ and $M_{22}$ are determined by the posterior mean of *M*_*w*_/*M*.

**Fig 6:**
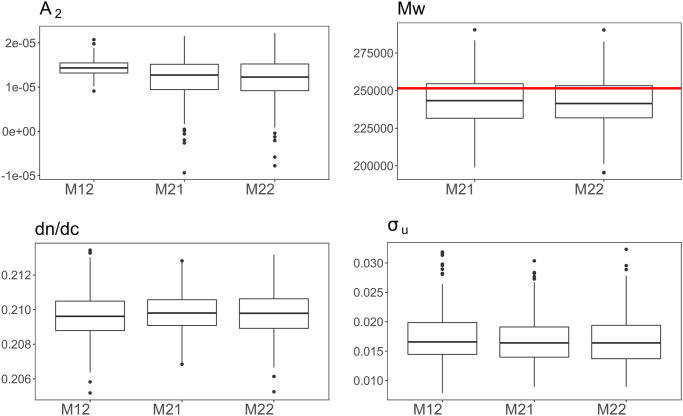
Boxplots of posterior samples of weight average molecular weight *M*_*w*_ and *A*_2_ for H*γ*S. The red horizontal line in the boxplot for *M*_*w*_ indicates the value of *M*_*w*_ under model $M_{12}$, that is, 12 × 20959.80 = 251517.6 g/mol.

Fig 5 presents the DIC values for the candidate oligomerization state models. We observe that *x* = 12 yields the smallest DIC value, which is similar to the DIC of $M_{21}$ and $M_{22}$. Fig 6 shows that $M_{12}$, $M_{21}$ and $M_{22}$ yield similar posterior median mass estimates (1.43, 1.22 and 1.29 × 10^−5^ mLmol/g^2^, respectively) and probabilities of being positive (1, 0.988 and 0.995, respectively) for *A*_2_, though the latter two models give wider posterior intervals. In addition, these models also yield similar inference on *M*_*w*_, suggesting the dodecamer (*x* = 12) might be the dominant structure in human *γ*S-crystallin solution under this solution condition (pH = 6.9, NaCl concentration: 100mM), with a nontrivial chance that decameric (*x* = 10), undecameric (*x* = 11) and tridecameric (*x* = 13) forms might exist as well. The model assessment parameters can be found in Section 3 in S1 File. Taken together, the combination of large *M*_*w*_ and positive *A*_2_ suggests a “self-avoiding cluster” model for H*γ*S, in which monomers interact attractively to form moderately sized oligomers, with the oligomers tending to repel one another (possibly due to selective exposure of less favorable interaction sites on the surface of the cluster, with sites favorable to surface interaction occupied by interactions with other cluster members). Such behavior may in part explain how *γ*S-crystallin manages the simultaneous functional requirements of high-density packing within the eye lens (necessary to provide the refractive index needed for lens function) and aggregation resistance (necessary for the lens to operate over the lifespan of the organism, given the absence of protein turnover in the mature lens): dodecameric packing is extremely space-efficient (allowing for high local concentration), while repulsion between dodecameric clusters avoids aggregation. If this hypothesized mechanism is correct, it is plausible that this pattern will be found in other lens crystallins. Alternatively, it may be that the observed dodecamers occur only in isolated H*γ*S, but are absent in the lens, which contains a mixture of structural and chaperone crystallins. These hypotheses would seem to be a fruitful target for future experimental research.

In passing, we note that when multiple competing models ($M_{1},\ldots ,M_{m}$) represent very different scientific mechanisms but yield similar performance in terms of the model selection criteria, Bayesian Model Averaging (BMA) [59] can be leveraged to incorporate the model uncertainty into the posterior inference of the physical quantities of interest (e.g., *A*_2_ in this analysis), which is another advantage of pursuing a Bayesian solution to this problem. Given the MCMC samples, the marginal likelihood $P(\text{data}|M_{j}),\;j=1,2,\ldots ,m$ required in BMA can be calculated by various different methods, including one-block Metropolis–Hastings method [60], power posteriors and thermodynamic integration [61, 62], etc. Although we do not pursue this here (since the high-probability models in this case are already in strong substantive agreement), this approach would be viable in cases where greater differences were observed in posterior estimates.

## Simulation study

A recurring theme in our analysis has been the importance of accounting for both uncertainty and measurement error in concentration. Here, we conduct a systematic simulation study to shed light on the impact of sample size and error control in concentration on inferential accuracy, thereby providing guidance for the design of future experiments. As *A*_2_ is the key physical quantity of interest in SLS experiments of the type discussed here, we focus on how well it can be estimated using the proposed Bayesian model. Specifically, the metrics for evaluating model performance are the bias of posterior means, the frequentist coverage and the width of posterior 95% credible intervals. As there are many different components of the proposed model, some of which are subject to physical constraints or the precision level of instruments—we fix those quantities at physically meaningful values and perform a full factorial design on the following four factors

1. *A*_2_ = ±0.01, ±0.001, ±0.0001, ±0.00001. These values cover the possible order of magnitude for *A*_2_ in most real-world proteins. As chosen in the case studies, we consider the flat prior $N(0,1^{2})$ (with respect to the scales of possible values of *A*_2_) across all simulation runs.
2.
$\sigma_{u}^{2}={\left(\text{log}\left(1+0.01\right)/1.96\right)}^{2},{\left(\text{log}\left(1+0.05\right)/1.96\right)}^{2},{\left(\text{log}\left(1+0.10\right)/1.96\right)}^{2},{\left(\text{log}\left(1+0.20\right)/1.96\right)}^{2}$ . These choices correspond to the possible range of percentage errors in concentration measurements, 1%, 5%, 10% and 20%, respectively.
3. Prior on $\sigma_{u}^{2}$:
  - Informative ($\sigma_{u}^{2}∼IG\left(1+\frac{1}{2+2\sigma_{u}^{2}},\frac{1}{2}\right)$): The informative prior is an Inverse-*χ*^2^ distribution with prior mean equal to corresponding true $\sigma_{u}^{2}$.
  - Intermediate informative ($\sigma_{u}^{2}∼IG\left(1,\sigma_{u}^{2}\right)$): intermediate informative prior is an Inverse-Gamma distribution that concentrates a considerable amount of its mass around true value of $\sigma_{u}^{2}$ while being fairly spread.
  - Weakly informative ($\sigma_{u}^{2}∼IG\left(1,{\left(\text{log}\left(1+0.40\right)/1.96\right)}^{2}\right)$): the weakly informative prior is an Inverse-Gamma distribution that concentrates most of its mass around a realistic upper bound (40% relative error) of the measurement errors in concentrations while being fairly spread.
  - No adjustment: Assuming the measured concentration is the true concentration, which can be viewed as a Bayesian analog to the bootstrapped regression modeling approach introduced in [19].
4. Number of experiment replicates (dictating the sample size): 1, 2, 5, 10.

We have a total of 8 × 4 × 4 × 4 = 512 settings, each of which is run for 100 replicates. All MCMC chains are run for 300000 iterations, and we store every 250th iteration of the last 100000 draws as posterior samples (the first 200000 draws are discarded as burn-in). The data are generated using the model described above, the concentration levels are set as those in Table 3, and the true values of $\sigma_{R}^{2}$ and $\sigma_{\Delta n}^{2}$ are fixed at the posterior mean reported in Table 4 to mimic the settings in real SLS experiments. Table 7 shows the ground truth values and priors for parameters that are unchanged across different experimental runs.

**Table 7:** True values and priors for parameters that are unchanged across different experimental runs. The symbol “-” means no prior assigned to the corresponding quantity (i.e., known constant that is not inferred using the model).

	True value	Prior
$\sigma_{R}^{2}$	1 × 10^−11^	*IG*(1, 10^−10^)
$\sigma_{\Delta n}^{2}$	2 × 10^−9^	*IG*(1, 10^−8^)
*dn*/*dc*	0.20	$N(0.195,0.005^{2})$
*n*_0_	1.33	-
λ	657 × 10^−7^	-
*M*	14307	-

Tables 8 and 9 show the relative bias of posterior mean estimates (i.e., $E(A_{2}-{\hat{A}}_{2})/A_{2}$, where ${\hat{A}}_{2}$ is the posterior mean estimate and *A*_2_ is the true value) and the frequentist coverage of 95% posterior intervals, respectively. Examination of the results leads to a number of practical insights that can inform experimental procedure (thus illustrating the value of statistical modeling to inform data collection practices):

- Estimating *A*_2_ accurately becomes harder as it decreases, and improper treatment of the concentration error or small sample size can lead to substantial bias. It is thus particularly important to attend to these issues when *A*_2_ is potentially low.
- Compared to models where measurement errors in concentration are adjusted for, the model with no adjustment leads to larger relative bias when the error in concentrations is large (> 10%) and the absolute value of true *A*_2_ is relatively large (⩾ 0.001).
- A major concern with not adjusting for measurement errors in concentration is the “no-adjustment” model’s inability to provide correct uncertainty estimates, that is, substantial undercoverage, given the error in concentration measurements are beyond 5%. This problem persists when the sample size is large.
- When the measurement errors are adjusted, upward biases are generally associated with negative *A*_2_, while downward biases are mainly associated with positive *A*_2_, regardless of other factors. It is also worth noting that we have slightly more difficulty in estimating negative *A*_2_ values compared to positive *A*_2_ values. We observe that, in general, the detection of repulsive effects is harder than that of attractive effects in dilute solution (a physical asymmetry that should be borne in mind e.g. when selecting sample sizes).
- Larger sample size improves performance (i.e., more replicates can help mitigate the bias), with diminishing returns for going beyond 5 replicates.
- A considerable gap exists between the performance of the weakly informative prior and the other two prior choices, while the gap between two other priors is often minimal. For robustness, we recommend the “intermediate informative” prior.
- Larger discrepancies between measured concentrations and the actual concentrations can lead to substantial bias, and such bias may persist even if we know its magnitude very well and do many replicates, especially when the absolute value of *A*_2_ is small. Therefore, *high precision in concentration measurements is crucial*. We suggest that experimenters use high-precision concentration measurement devices, reduce the errors in liquid handling, and more importantly, measure concentrations both before and after the LS and RI experiments, if possible.

**Table 8:** Relative bias of posterior mean estimates of *A*_2_ under different settings.

True values			Informative				Intermediate informative				Weakly informative				No adjustment			
|*A*_2_|	*A*_2_	concentration errors in(%)	1	2	5	10	1	2	5	10	1	2	5	10	1	2	5	10
0.01	0.01	1.00	0.01	0.00	0.00	0.00	0.01	0.00	0.00	0.00	-0.00	-0.00	-0.00	-0.00	0.01	0.00	0.00	-0.00
0.01	0.01	5.00	0.01	0.00	0.00	0.00	0.01	0.00	0.00	0.00	-0.00	-0.00	-0.00	-0.00	0.01	0.00	0.01	0.00
0.01	0.01	10.00	0.00	0.00	0.00	0.00	0.01	0.00	0.00	0.00	-0.00	-0.00	-0.00	-0.00	0.02	0.01	0.02	0.00
0.01	0.01	20.00	0.00	-0.00	0.00	-0.00	0.00	-0.00	0.00	-0.00	-0.00	-0.00	-0.00	-0.00	0.04	0.03	0.04	0.02
0.01	-0.01	1.00	0.01	0.00	0.00	-0.00	0.01	0.00	0.00	-0.00	0.00	0.00	0.00	-0.00	0.01	0.00	0.00	-0.00
0.01	-0.01	5.00	0.01	0.00	0.00	-0.00	0.01	0.00	0.00	-0.00	0.00	0.00	0.00	-0.00	0.01	0.01	0.01	0.00
0.01	-0.01	10.00	0.01	0.00	0.00	-0.00	0.01	0.00	0.00	-0.00	0.00	0.00	0.00	-0.00	0.02	0.01	0.02	0.00
0.01	-0.01	20.00	0.01	0.00	0.00	-0.00	0.01	0.00	0.00	-0.00	0.00	0.00	0.00	-0.00	0.05	0.04	0.04	0.02
0.001	0.001	1.00	0.00	-0.00	0.00	-0.00	0.00	-0.00	0.00	-0.00	-0.01	-0.01	-0.00	-0.00	0.00	-0.00	0.00	-0.00
0.001	0.001	5.00	0.00	-0.00	0.00	0.00	0.00	-0.00	0.00	0.00	-0.01	-0.01	-0.00	-0.00	0.00	-0.00	0.00	0.00
0.001	0.001	10.00	0.00	-0.00	0.00	0.00	0.00	-0.00	0.00	0.00	-0.01	-0.01	-0.00	-0.00	0.00	0.00	0.01	0.00
0.001	0.001	20.00	-0.00	-0.01	0.00	-0.00	-0.00	-0.01	0.00	-0.00	-0.01	-0.01	-0.00	-0.00	0.01	0.01	0.02	0.01
0.001	-0.001	1.00	0.01	0.01	0.00	0.00	0.01	0.01	0.00	0.00	0.04	0.03	0.01	-0.00	0.01	0.01	0.00	0.00
0.001	-0.001	5.00	0.02	0.02	0.01	0.00	0.02	0.02	0.01	0.00	0.04	0.03	0.01	-0.00	0.02	0.01	0.01	0.00
0.001	-0.001	10.00	0.04	0.03	0.02	0.00	0.04	0.03	0.02	0.00	0.04	0.04	0.01	-0.00	0.03	0.02	0.03	0.00
0.001	-0.001	20.00	0.05	0.04	0.02	0.00	0.05	0.04	0.02	0.00	0.04	0.04	0.02	0.00	0.07	0.05	0.06	0.02
0.0001	0.0001	1.00	-0.04	-0.03	-0.00	-0.00	-0.05	-0.04	-0.00	-0.00	-0.18	-0.07	0.06	0.04	-0.04	-0.03	-0.00	-0.00
0.0001	0.0001	5.00	-0.10	-0.06	-0.03	-0.01	-0.09	-0.06	-0.03	-0.01	-0.17	-0.08	0.04	0.04	-0.06	-0.04	-0.02	-0.00
0.0001	0.0001	10.00	-0.17	-0.11	-0.07	-0.02	-0.16	-0.10	-0.07	-0.02	-0.18	-0.10	0.00	0.03	-0.10	-0.07	-0.05	-0.00
0.0001	0.0001	20.00	-0.28	-0.21	-0.15	-0.06	-0.27	-0.20	-0.16	-0.06	-0.22	-0.14	-0.08	-0.01	-0.19	-0.13	-0.11	-0.01
0.0001	-0.0001	1.00	0.06	0.04	0.01	0.00	0.06	0.04	0.01	0.00	0.25	0.17	-0.02	-0.04	0.05	0.04	0.01	0.00
0.0001	-0.0001	5.00	0.12	0.08	0.04	0.01	0.12	0.07	0.04	0.01	0.25	0.18	0.00	-0.04	0.08	0.05	0.04	0.00
0.0001	-0.0001	10.00	0.23	0.16	0.10	0.02	0.21	0.15	0.10	0.03	0.26	0.20	0.05	-0.02	0.14	0.09	0.09	0.01
0.0001	-0.0001	20.00	0.37	0.29	0.21	0.07	0.36	0.29	0.21	0.07	0.29	0.26	0.15	0.03	0.29	0.20	0.21	0.04
0.00001	0.00001	1.00	-0.49	-0.36	-0.06	-0.03	-0.51	-0.37	-0.06	-0.03	-2.15	-1.17	0.42	0.46	-0.47	-0.35	-0.05	-0.03
0.00001	0.00001	5.00	-1.09	-0.70	-0.35	-0.08	-1.04	-0.65	-0.34	-0.07	-2.14	-1.23	0.19	0.42	-0.67	-0.47	-0.29	-0.01
0.00001	0.00001	10.00	-1.99	-1.30	-0.84	-0.23	-1.86	-1.22	-0.85	-0.24	-2.19	-1.47	-0.21	0.27	-1.17	-0.76	-0.65	-0.04
0.00001	0.00001	20.00	-3.25	-2.48	-1.84	-0.65	-3.16	-2.44	-1.89	-0.66	-2.53	-2.00	-1.20	-0.19	-2.35	-1.59	-1.53	-0.21
0.00001	-0.00001	1.00	0.51	0.37	0.06	0.03	0.53	0.38	0.06	0.03	2.20	1.33	-0.38	-0.46	0.47	0.35	0.06	0.03
0.00001	-0.00001	5.00	1.12	0.71	0.37	0.08	1.05	0.66	0.36	0.08	2.25	1.36	-0.13	-0.41	0.68	0.47	0.30	0.01
0.00001	-0.00001	10.00	2.05	1.35	0.87	0.24	1.91	1.24	0.89	0.25	2.30	1.53	0.28	-0.26	1.23	0.79	0.69	0.05
0.00001	-0.00001	20.00	3.32	2.55	1.90	0.66	3.24	2.51	1.93	0.68	2.56	2.12	1.25	0.20	2.45	1.68	1.64	0.26

**Table 9:** Coverage rates of 95% credible intervals for *A*_2_ under different settings.

True values			Informative				Intermediate informative				Weakly informative				No adjustment			
|*A*_2_|	*A*_2_	concentration errors in(%)	1	2	5	10	1	2	5	10	1	2	5	10	1	2	5	10
0.01	0.01	1.00	1.00	0.99	0.95	0.94	1.00	0.99	0.95	0.96	1.00	1.00	0.96	0.95	1.00	0.99	0.93	0.98
0.01	0.01	5.00	1.00	0.98	0.97	0.96	1.00	0.99	0.97	0.95	1.00	1.00	0.97	0.97	0.80	0.79	0.82	0.88
0.01	0.01	10.00	1.00	1.00	0.97	0.94	1.00	1.00	0.97	0.94	1.00	1.00	0.97	0.93	0.72	0.74	0.76	0.83
0.01	0.01	20.00	0.99	1.00	0.97	0.93	0.99	1.00	0.97	0.94	1.00	0.99	0.97	0.94	0.66	0.68	0.66	0.77
0.01	-0.01	1.00	1.00	1.00	0.95	0.99	1.00	0.99	0.95	0.98	1.00	1.00	0.95	0.98	0.98	0.99	0.93	0.97
0.01	-0.01	5.00	1.00	0.99	0.94	0.99	1.00	1.00	0.95	0.98	1.00	1.00	0.94	0.99	0.77	0.74	0.81	0.88
0.01	-0.01	10.00	1.00	1.00	0.94	0.99	1.00	0.99	0.94	0.99	1.00	1.00	0.95	0.99	0.72	0.73	0.76	0.83
0.01	-0.01	20.00	1.00	1.00	0.93	0.97	1.00	1.00	0.92	0.97	1.00	1.00	0.94	0.98	0.65	0.69	0.67	0.78
0.001	0.001	1.00	0.99	0.97	0.95	0.94	1.00	0.98	0.93	0.93	1.00	1.00	1.00	0.99	0.99	0.98	0.92	0.90
0.001	0.001	5.00	0.98	0.99	0.93	0.93	0.98	0.97	0.94	0.93	1.00	1.00	1.00	1.00	0.92	0.85	0.72	0.76
0.001	0.001	10.00	0.97	0.98	0.94	0.94	0.97	0.96	0.93	0.95	1.00	1.00	1.00	0.98	0.77	0.67	0.62	0.70
0.001	0.001	20.00	0.97	0.94	0.93	0.95	0.95	0.92	0.94	0.95	1.00	1.00	0.98	0.96	0.65	0.62	0.51	0.60
0.001	-0.001	1.00	1.00	0.99	0.95	0.99	1.00	0.98	0.96	0.99	1.00	1.00	0.99	1.00	0.99	0.95	0.95	0.96
0.001	-0.001	5.00	1.00	0.99	0.92	0.98	0.98	0.97	0.90	0.98	1.00	1.00	1.00	1.00	0.81	0.75	0.82	0.85
0.001	-0.001	10.00	1.00	0.99	0.92	0.98	0.98	0.98	0.91	0.98	1.00	1.00	0.98	0.99	0.70	0.72	0.77	0.83
0.001	-0.001	20.00	1.00	0.99	0.93	0.99	0.99	0.98	0.93	0.99	1.00	1.00	0.96	0.99	0.69	0.66	0.73	0.80
0.0001	0.0001	1.00	1.00	0.98	0.97	0.97	1.00	0.98	0.97	0.96	1.00	1.00	1.00	1.00	1.00	0.98	0.97	0.95
0.0001	0.0001	5.00	0.99	0.98	0.94	0.99	0.99	0.95	0.94	0.96	1.00	1.00	1.00	1.00	0.97	0.89	0.89	0.95
0.0001	0.0001	10.00	0.98	0.95	0.92	0.98	0.97	0.91	0.91	0.97	1.00	1.00	1.00	1.00	0.85	0.81	0.85	0.87
0.0001	0.0001	20.00	0.99	0.94	0.91	0.96	0.97	0.90	0.88	0.96	1.00	1.00	0.99	0.99	0.75	0.75	0.81	0.84
0.0001	-0.0001	1.00	1.00	0.98	0.98	0.97	1.00	0.98	0.98	0.96	1.00	1.00	1.00	1.00	1.00	0.97	0.96	0.96
0.0001	-0.0001	5.00	0.99	0.98	0.93	0.98	0.99	0.95	0.92	0.97	1.00	1.00	1.00	1.00	0.92	0.83	0.88	0.89
0.0001	-0.0001	10.00	0.99	0.95	0.91	0.98	0.97	0.91	0.90	0.98	1.00	1.00	1.00	1.00	0.77	0.76	0.84	0.84
0.0001	-0.0001	20.00	0.97	0.93	0.89	0.96	0.95	0.93	0.89	0.96	1.00	1.00	0.98	1.00	0.72	0.71	0.77	0.83
0.00001	0.00001	1.00	1.00	0.98	0.97	0.97	1.00	0.98	0.98	0.96	1.00	1.00	1.00	1.00	1.00	0.97	0.97	0.96
0.00001	0.00001	5.00	1.00	0.99	0.94	0.98	0.99	0.94	0.93	0.96	1.00	1.00	1.00	1.00	0.94	0.85	0.90	0.93
0.00001	0.00001	10.00	0.99	0.94	0.89	0.97	0.97	0.92	0.87	0.97	1.00	1.00	1.00	1.00	0.82	0.78	0.84	0.84
0.00001	0.00001	20.00	0.99	0.92	0.90	0.96	0.98	0.93	0.91	0.96	1.00	1.00	0.99	0.99	0.75	0.72	0.76	0.84
0.00001	-0.00001	1.00	1.00	0.98	0.98	0.96	1.00	0.98	0.97	0.97	1.00	1.00	1.00	1.00	1.00	0.98	0.97	0.97
0.00001	-0.00001	5.00	1.00	0.99	0.94	0.99	1.00	0.97	0.93	0.97	1.00	1.00	1.00	1.00	0.92	0.83	0.87	0.91
0.00001	-0.00001	10.00	0.99	0.94	0.90	0.98	0.96	0.93	0.88	0.97	1.00	1.00	1.00	1.00	0.80	0.79	0.85	0.86
0.00001	-0.00001	20.00	0.97	0.94	0.90	0.97	0.96	0.92	0.90	0.96	1.00	1.00	0.99	1.00	0.74	0.72	0.76	0.82

## Discussion

### Considerations for experimental procedure

As illustrated by the results of our simulation study, controlling errors in concentrations is the key to accurate static light scattering experiments. Naive methods with no adjustment for concentration errors can lead to reliable estimates when the relative error in concentration is extremely small (e.g. 1%). As the concentration errors become larger, accurate results require at minimum that we have some knowledge about the possible range of the measurement errors in concentration, and account for such errors in the model; further, larger sample sizes (e.g., more experimental replicates) are required to mitigate the bias caused by the concentration errors. As this suggests, our findings *strongly underscore the importance of minimizing concentration errors when performing SLS experiments*, and motivate the development of improved protocols to control this source of error. However, we do find that good results can be obtained with non-vanishing levels of concentration error, so long as an appropriate error model is used and adequate numbers of replicates are performed. While error reduction via improved procedure is always a priority, building in replicates and avoiding “no-adjustment” models is recommended in practice.

## Conclusion

In this article, we proposed a novel Bayesian model for static light scattering (SLS) data that is sufficiently flexible to accommodate the complex relationship between physical quantities and their measurements, and to account for measurement errors. We ran simulation studies to gain insights about how measurement errors and sample size can affect the estimation of the second virial coefficient, and converted these insights into actionable guidance for future experiments. With the proposed model, we studied the protein aggregation behavior of two important proteins, lysozyme and human *γ*S-crystallin, in the former case identifying the conditions under which monomers transition from repulsive to attractive interactions, and in the second case showing the presence of a distinctive “self-avoiding cluster” structure in which monomers form oligomers of approximately dodecameric order which then interact repulsively. Facilitating this was a protocol for cleaning and pre-processing SLS data, which provides a largely automated way to remove common artifacts and detect problems in data acquisition.

This article demonstrates the great value of Bayesian statistics in advancing data analysis within the biophysical context. Firstly, Bayesian analysis provides a principled way to update our beliefs about physical quantities using a combination of existing knowledge and experimental data. Secondly, though error modeling from a frequentist perspective is powerful, it can suffer from identitifiability problems if the error mechanism is not precisely known, or if certain classes of errors cannot be strictly ruled out. In contrast, Bayesian treatments are less sensitive to such difficulties so long as the posteriors remain characterizable, and informative priors can aid in filling in information that the data alone cannot supply. As considerable background information is often possible in biophysical settings, this is a natural context for employing informative Bayesian analysis. Thirdly, the Bayesian perspective can provide fully probabilistic answers to many scientific questions of interest, e.g, questions such as “what is the probability of *A*_2_ being positive given the experimental data?” This advantage is highly valuable for problems such as *A*_2_ estimation, where measurement is inherently difficult and residual uncertainty is expected to be large. Last but not least, continued advances in computational techniques mean that the “Bayesian crank” can be easily implemented using various freely available software packages, making it easier to supply solutions to practitioners without requiring them to be experts in e.g. MCMC simulation.

Given a powerful and flexible statistical model for the analysis of static light scattering data, researchers will be able to gain better understanding of the mechanisms governing protein aggregation. Such advances have the potential to inform areas such as medical research to develop better treatments for diseases such as Alzheimer’s and Parkinson’s Diseases, which are caused by protein aggregation.

In closing, we comment on four potential directions for future work. First of all, we only work with the LS readings from angle *θ* = 90° in this analysis. Incorporating additional angles where available may improve precision, although it then becomes necessary to account for additional sources of error associated with mechanisms such as differences in detector alignment or sensitivity. Secondly, this work is concerned with small proteins with *P*(*r*_*g*_, *θ*) ≈ 1, and it is natural to consider extending our approach to large particles. Such an extension also requires further investigation on the use of readings from angles other than *θ* = 90°. Thirdly, motivated by the need to inform simulation-based work on protein aggregation, it would be interesting to consider whether higher-order virial coefficients could be inferred. While present experimental methodology lacks the precision required for such analyses in settings like those studied here, future developments may remove this barrier. Finally, the concentration levels in the experiments analyzed in this paper are chosen based on the experimenters’ heuristics and the difficulty posed by different concentration ranges for sample preparation. It seems natural to attempt to improve on this by setting concentrations using sampling design theories for regression models (see, e.g., [63–66]), potentially leading to more efficient experiments with similar inferential power.

## Supporting information

S1 File The supplementary file contains details of (1) a systematic data cleaning algorithm for removing experimental artifacts; (2) posterior predictive assessments for two case studies presented; (3) a table showing relative width (average width / true |*A*_2_| value) of 95% posterior credible intervals of *A*_2_ in the simulation study. R and JAGS codes along with the data for the computations in this paper are available from https://github.com/fyin-stats/bayes-light-scattering.
(PDF)
