# Supplementary material for: Bayesian analysis of static light scattering data for globular proteins
Source: PLoS One. 2021 Oct 14;16(10):e0258429. doi: 10.1371/journal.pone.0258429 (PMC8516215; doi:10.1371/journal.pone.0258429)
Supplement: S1 File — R and JAGS codes along with the data for the computations in this paper are available from https://github.com/fyin-stats/bayes-light-scattering. (PDF) [file pone.0258429.s001.pdf]

Web-based Supplementary File for "Bayesian Analysis of Static Light Scattering Data for Globular Proteins"

1 Data Cleaning and Preparation

Here we describe procedures involved in cleaning raw SLS data and preparing it for use. These procedures were employed for the data cases examined in this work, but are also suggested as a practical workflow for handling data of this kind more generally. Although our emphasis is on data processing, some comments are also made regarding experimental procedure; it is helpful for the analyst to be aware of these issues both to avoid misinterpretation of experimental artifacts (e.g., spurious transient signals due to air bubbles) and to provide guidance to experimental collaborators regarding elements of procedure that may complicate subsequent data analysis.

To obtain the data used in this paper, we performed static light scattering experiments on aqueous solutions of hen egg white lysozyme and human γS-crystallin, using a combination of a Dawn HELEOS multi-angle light scattering (LS) detector and an Optilab rEX refractive index (RI) detector from Wyatt Technology (Santa Barbara, CA). Figure S1 shows a flowchart for an SLS experiment. Using batch-mode injections, prepared samples were injected through a flow cell using a syringe pump starting with buffer only, followed by lysozyme samples (lowest to highest concentration), and finally ending with buffer only for baseline correction. Filters (0.1μm) were used with an aim to enhance monodispersity as samples are injected through the flow cell. Batch-mode collection does introduce potential artifacts causing some inconsistencies in the scattering intensities. These artifacts are results of air bubbles or back pressure from the injection of a new sample during data collection. In this section, we propose an automatic procedure for removing these common and unavoidable artifacts, which prove to greatly facilitate the SLS data preparation process.

1.1 Removal of Experimental Artifacts

As noted above, the primary source of experimental artifacts for both light scattering and refractive index measurements are transient changes in signal due to changes in concentration (during the transition from one concentration to another) and/or the presence of air bubbles in the syringe or the flow cell. In practice, these manifest as large deviations in the input series that are easily noted on inspection of the raw data (see left panel of Figure 2in the main text). To detect and remove these contaminated observations, we employ a local estimate of the absolute time derivative of the measured signal as a notion of measurement "stability." Specifically, let $y_1, \ldots, y_m$ be a sequence of raw scattering or refractive index measurements at times $t_1, \ldots, t_m$. We then estimate the instability of measurement y_i by

$$\hat{s}_i(y,t) = \left[\frac{|y_{i+1} - y_i|}{t_{i+1} - t_i} + \frac{|y_i - y_{i-1}|}{t_i - t_{i-1}} \right] /2,$$

where $|\cdot|$ indicates the absolute value.

To trim transients from the data set, we remove all observations y_i such that

$$\frac{\hat{s}_i(y,t) - Q_{50}\left(\hat{s}(y,t)\right)}{\mathrm{IQR}\left(\hat{s}(y,t)\right)} > \tau,$$

where Q_{50} is the 0.5 quantile (i.e., sample median), IQR is the inter-quartile range, and τ is a user-selected threshold. We typically select $\tau = 1$, which removes all observations whose instability is estimated to be more than one IQR above the median for that time series; in the event that inspection of the trimmed series reveals some "flyaway" points remaining, smaller values of τ may be necessary.

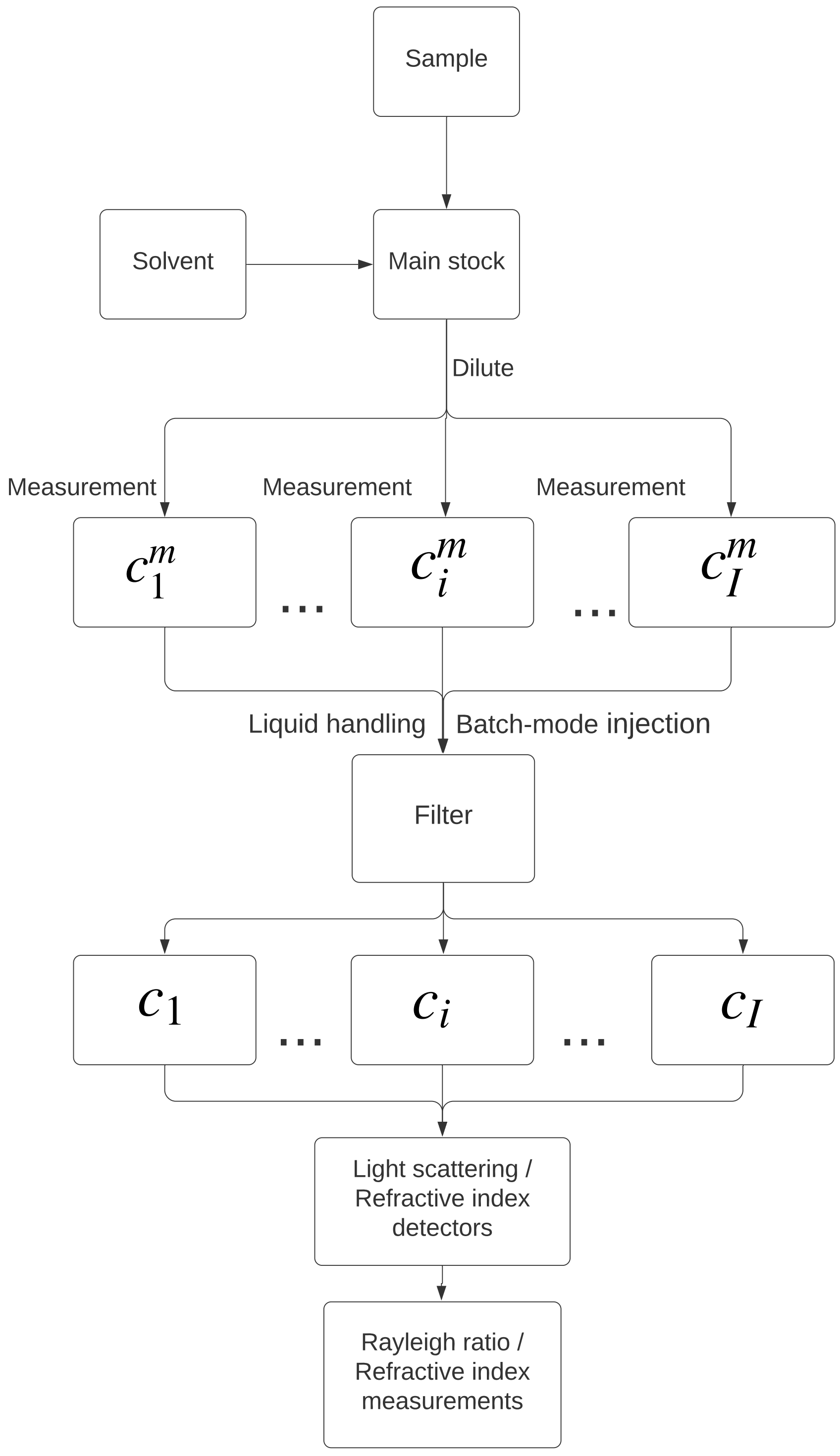

Figure S1: Flowchart showing SLS experimental procedure; labeled quantities are employed in subsequent analyses.

An example of the impact of trimming on raw data series is shown in the right panel of Figure 2 from the main text. As can be seen, the above criteria have successfully removed most experimental artifacts, leaving a stable interval of points associated with each concentration. We have found that this approach (with $\tau = 1$) typically works quite well, with reductions of τ to 0.5 or 0 usually sufficing to remove artifacts in difficult cases. Occasionally, especially dramatic transient fluctuations will yield small clusters of points between concentration levels that are not caught by $\hat{s}$ directly (due the to the fact that the signal fluctuation temporarily "pauses"). In these cases, we have found that applying a mild moving average smoothing to $\hat{s}$ by using $\hat{s}'_i(y,t) = (\hat{s}_{i-1}(y,t) + \hat{s}_i(y,t) + \hat{s}_{i+1}(y,t))/3$ in place of $\hat{s}_(y,t)$ readily corrects the problem.

1.2 Preparation of Raw Measurements

After removing the experimental artifacts with the procedure described in 1.1, we propose to match the observations with the concentration levels using a clustering algorithm, and then summarize these observations using a robust estimator (e.g., the median of the trimmed observations) to assign a single refractive index value and a single Rayleigh ratio value to each concentration level.

1.2.1 Clustering of Measurements by Concentration

Although manual identification of data points associated with each concentration condition is possible, we instead automate the process using a constrained agglomerative clustering algorithm. We initialize the algorithm by taking each set of raw measurements (post-filtering) to be an initial "cluster;" we observe that each such measurement is associated with a particular time point, which we exploit in the agglomeration process. In particular, we consider at each iteration the merger of each current cluster with its immediate temporal neighbors (i.e., the clusters immediately prior and immediately following it in temporal order). The cost of merging two clusters is determined as follows. First, the merge penalty for two points is taken to be the median absolute difference between all measurements taken on those points (i.e., if scattering at k angles is observed, the median of the absolute differences over the k angles gives the partial penalty associated with placing these two points within the same cluster. The merge cost for two clusters is then defined to be the median of the pointwise merge penalties for all point pairs in the respective clusters. At each stage in the clustering process, the merge cost for each pair of temporally adjacent clusters is computed, and the lowest cost merge is performed. This process is repeated until the number of clusters is equal to the number of design concentration conditions (plus a concentration zero baseline immediately before and after the non-zero concentrations).

This algorithm is simple to implement and produces solutions with the desired properties of temporal contiguity (a basic feature of the experimental design), repeatability, and optimality with respect to merge cost. Use of the double median merge cost minimizes the impact of any unstable measurements not removed during the filtering stage on the clustering solution.

1.2.2 Initial Scattering and Refractive Index Measurements

Given the assignment of (filtered) raw scattering and refractive index measurements to concentration levels, we then obtain initial estimates of $R(\theta, c)$ and n_c by taking the sample median of the raw measurements in each cluster. Our choice of the sample median is motivated by robustness to remaining unstable measurements; since the number of raw observations per concentration is generally large (e.g., 50-300), efficiency is not a substantial concern. To obtain a robust initial estimate of the variability of each point estimate, we employ the nonparametric confidence intervals of (Gibbons and Chakraborti, 2011, eq. 5.4.10). (Note that, at this stage in our analysis, we seek an estimate of the variability in the measurement, *not* the underlying parameter. These are used to set priors for measurement errors within the subsequent stage of analysis.)

1.3 Additional Considerations for Practice

To date, we have found the above approach to work well in handling refractive index and light scattering data taken over a range of concentrations, on multiple instruments. Typically, little or no manual intervention is required. However, it is important for the analyst to visually inspect the clustering solution and initial estimates for both scattering and refractive index series to ensure that: (1) unstable observations were

properly removed; (2) clustered observations are sensible (and do not e.g. group together observations taken at different concentrations); and (3) initial scattering/refractive index measurements properly represent the central tendencies of measurements at their respective concentration levels. In our experience, problems with this process invariably result from poor filtering of unstable points. This can be rectified by modifying the choice of τ and/or applying moving average smoothing to $\hat{s}$ as described in Section 1.1.

Although our focus here is on data analysis, some considerations relating to experimental conditions should also be borne in mind. As we have emphasized above (and show in the main text), SLS experiments and resulting inference are more sensitive to concentration than many biophysical experiments, and hence precision in sample preparation and liquid handling are a must. We have also observed that, with multi-angle detectors, it is not uncommon for some detectors to function poorly or to have subtle hardware defects such as loose collimators that induce "drift" in measurements over the course of the experiment (observable by inspection as large and often dynamic changes in signal relative to other detectors under simultaneous data acquisition). Inspection of raw and processed data for bad detectors (and their correction or removal) is hence recommended. Likewise, it is recommended that the linearity of the RI signal with respect to concentration be verified; we have observed that some refractometers intended for use with SLS experiments will give erroneous readings for proteins with anomalously high dn/dc values, especially at high concentrations, manifesting as large, unphysical, and non-monotone changes in measured RI when the RI exceeds a threshold value. Removal of RI measurements for these conditions is then essential for accurate analysis. Another important experimental parameter is the temperature, which may produce apparent differences in the refractive index data if uncontrolled (Abbate et al., 1978; Tan and Huang, 2015).

2 Model assessment for case study on Lysozyme

Table S1 and S2 display the posterior predictive p-values of Rayleigh ratio and Δn readings. Most of the predictive p-values are within the range of roughly 0.25 to 0.75, suggesting the model provides a good fit to the observed data, especially for Δn readings. It is worth noting that the posterior predictive p-values for Rayleigh ratio readings are closer to 0.50 for large concentration levels, and the fit for two experiments (pH = 4.7, NaCl : 150mM; pH = 6.9, NaCl = 250mM) are slightly worse compared to others. The latter may suggest the presence of unobserved factors associated with experimental procedure affecting these data points. However, the data are nevertheless quite compatible with what would be expected under the model.

Table S1: Posterior predictive p-values for Rayleigh ratio measurements under $\mathcal{M}_1$.

NaCl (mM)	50	75	100	125	150	300	50	75	100	125	150	200	250	300
Level (i=)			pH = 4.7							pH = 6.9				
1	0.613	0.547	0.565	0.398	0.013	0.468	0.573	0.560	0.502	0.133	0.435	0.580	0.335	0.395
2	0.605	0.610	0.630	0.435	0.007	0.515	0.490	0.588	0.490	0.122	0.443	0.525	0.185	0.463
3	0.760	0.657	0.682	0.502	0.022	0.485	0.487	0.530	0.398	0.113	0.450	0.450	0.133	0.453
4	0.805	0.740	0.760	0.598	0.068	0.590	0.495	0.410	0.383	0.128	0.372	0.380	0.128	0.460
5	0.890	0.780	0.738	0.677	0.140	0.527	0.570	0.335	0.335	0.128	0.432	0.285	0.077	0.435
6	0.925	0.843	0.787	0.657	0.172	0.417	0.655	0.300	0.247	0.182	0.463	0.270	0.037	0.440
7	0.955	0.863	0.835	0.665	0.255	0.448	0.645	0.292	0.270	0.250	0.510	0.195	0.018	0.378
8	0.985	0.895	0.790	0.757	0.020	0.465	0.760	0.280	0.180	0.338	0.580	0.177	0.013	0.340
9	0.865	0.790	0.688	0.505	0.395	0.550	0.730	0.635	0.545	0.395	0.375	0.475	0.492	0.385
10	0.873	0.630	0.640	0.522	0.417	0.463	0.575	0.665	0.568	0.445	0.490	0.507	0.448	0.338
11	0.682	0.613	0.540	0.487	0.510	0.422	0.420	0.593	0.545	0.440	0.485	0.487	0.415	0.448
12	0.487	0.345	0.547	0.448	0.497	0.450	0.482	0.570	0.525	0.568	0.470	0.512	0.370	0.502
13	0.302	0.330	0.407	0.453	0.588	0.657	0.480	0.407	0.472	0.560	0.527	0.545	0.530	0.578
14	0.115	0.330	0.328	0.502	0.693	0.435	0.420	0.425	0.455	0.555	0.555	0.485	0.787	0.662

3 Model assessment for case study on human γS-crystallin

Table S3 and S4 show the posterior predictive p-values for models $\mathcal{M}_{21}$, $\mathcal{M}_{22}$ and $\mathcal{M}_{12}$, aggregated over replicates, for the Rayeligh ratio and Δn readings. These p-values are close to 0.5.

Table S2: Posterior predictive p-values for Δn measurements under $\mathcal{M}_1$.

NaCl (mM)	50	75	100	125	150	300	50	75	100	125	150	200	250	300
Level (i=)			pH = 4.7							pH = 6.9				
1	0.517	0.573	0.635	0.562	0.435	0.450	0.620	0.698	0.615	0.430	0.338	0.595	0.492	0.235
2	0.357	0.557	0.542	0.480	0.270	0.422	0.468	0.820	0.573	0.367	0.280	0.522	0.195	0.400
3	0.378	0.480	0.475	0.545	0.385	0.448	0.547	0.728	0.570	0.410	0.328	0.578	0.407	0.468
4	0.407	0.482	0.527	0.415	0.427	0.450	0.545	0.650	0.585	0.407	0.350	0.570	0.627	0.390
5	0.390	0.502	0.460	0.448	0.492	0.430	0.510	0.662	0.627	0.492	0.380	0.532	0.680	0.470
6	0.450	0.455	0.450	0.482	0.515	0.480	0.522	0.610	0.603	0.472	0.422	0.570	0.720	0.475
7	0.405	0.450	0.443	0.435	0.510	0.438	0.465	0.608	0.650	0.417	0.385	0.623	0.720	0.477
8	0.438	0.500	0.450	0.367	0.588	0.512	0.512	0.608	0.635	0.463	0.415	0.618	0.770	0.510

Table S3: Posterior predictive p-values for Rayleigh ratio measurements under $\mathcal{M}_{21}$, $\mathcal{M}_{22}$ and $\mathcal{M}_{12}$.

Level (i=)	$\mathcal{M}_{21}$	$\mathcal{M}_{22}$	$\mathcal{M}_{12}$
1	0.532	0.532	0.542
2	0.511	0.488	0.497
3	0.519	0.540	0.541
4	0.585	0.545	0.545
5	0.620	0.588	0.594
6	0.526	0.507	0.532
7	0.607	0.553	0.570
8	0.620	0.568	0.572
9	0.595	0.579	0.566
10	0.602	0.578	0.568
11	0.570	0.575	0.573
12	0.559	0.551	0.542
13	0.488	0.459	0.500
14	0.479	0.499	0.521

Table S4: Posterior predictive p-values for Δn measurements under $\mathcal{M}_{21}$, $\mathcal{M}_{22}$ and $\mathcal{M}_{12}$.

Level (i=)	$\mathcal{M}_{21}$	$\mathcal{M}_{22}$	$\mathcal{M}_{12}$
1	0.356	0.376	0.374
2	0.352	0.349	0.355
3	0.393	0.386	0.371
4	0.357	0.358	0.316
5	0.427	0.418	0.412
6	0.469	0.463	0.473
7	0.473	0.484	0.479
8	0.623	0.612	0.603
9	0.518	0.552	0.550
10	0.579	0.583	0.573
11	0.444	0.438	0.430
12	0.389	0.393	0.376

4 Table for relative width (average width / true $|A_2|$ value) of 95% posterior credible intervals of A_2

Table S5: Relative width (average width / true $|A_2|$ value) of 95% posterior credible intervals of A_2 under different settings.

True values			Informative				Intermediate informative				Weakly informative				No adjustment			
$\|A_2\|$	A_2	concentration errors in(%)	1	2	5	10	1	2	5	10	1	2	5	10	1	2	5	10
0.01	0.01	1.00	0.06	0.04	0.02	0.01	0.06	0.04	0.02	0.01	0.09	0.05	0.02	0.02	0.07	0.04	0.02	0.02
0.01	0.01	5.00	0.07	0.04	0.02	0.02	0.07	0.05	0.02	0.02	0.09	0.05	0.02	0.02	0.09	0.07	0.04	0.03
0.01	0.01	10.00	0.08	0.05	0.02	0.02	0.08	0.05	0.02	0.02	0.09	0.05	0.03	0.02	0.14	0.11	0.07	0.05
0.01	0.01	20.00	0.09	0.05	0.03	0.02	0.09	0.05	0.03	0.02	0.09	0.05	0.03	0.02	0.22	0.18	0.13	0.09
0.01	-0.01	1.00	0.08	0.05	0.03	0.02	0.08	0.05	0.03	0.02	0.11	0.06	0.03	0.02	0.08	0.05	0.03	0.02
0.01	-0.01	5.00	0.10	0.06	0.03	0.02	0.10	0.06	0.03	0.02	0.11	0.06	0.03	0.02	0.11	0.08	0.05	0.03
0.01	-0.01	10.00	0.11	0.06	0.03	0.02	0.11	0.06	0.03	0.02	0.11	0.06	0.03	0.02	0.16	0.12	0.08	0.06
0.01	-0.01	20.00	0.11	0.07	0.03	0.02	0.11	0.07	0.03	0.02	0.11	0.06	0.03	0.02	0.25	0.20	0.15	0.11
0.001	0.001	1.00	0.05	0.03	0.02	0.01	0.05	0.03	0.02	0.01	0.24	0.13	0.06	0.03	0.05	0.03	0.02	0.01
0.001	0.001	5.00	0.08	0.05	0.03	0.02	0.08	0.05	0.03	0.02	0.24	0.13	0.06	0.03	0.06	0.04	0.02	0.01
0.001	0.001	10.00	0.11	0.07	0.04	0.03	0.11	0.07	0.04	0.03	0.24	0.14	0.07	0.04	0.07	0.05	0.03	0.02
0.001	0.001	20.00	0.16	0.11	0.06	0.05	0.16	0.10	0.06	0.04	0.25	0.15	0.08	0.05	0.11	0.08	0.06	0.04
0.001	-0.001	1.00	0.14	0.09	0.05	0.03	0.14	0.09	0.05	0.03	0.46	0.29	0.16	0.09	0.13	0.08	0.05	0.03
0.001	-0.001	5.00	0.23	0.16	0.10	0.06	0.23	0.16	0.09	0.06	0.46	0.29	0.16	0.10	0.18	0.12	0.08	0.05
0.001	-0.001	10.00	0.32	0.23	0.14	0.09	0.32	0.22	0.13	0.09	0.46	0.30	0.17	0.11	0.25	0.19	0.13	0.09
0.001	-0.001	20.00	0.41	0.28	0.17	0.12	0.41	0.28	0.17	0.11	0.47	0.30	0.18	0.12	0.39	0.31	0.23	0.17
0.0001	0.0001	1.00	0.71	0.43	0.24	0.16	0.72	0.44	0.24	0.16	2.35	1.55	0.78	0.45	0.70	0.42	0.23	0.16
0.0001	0.0001	5.00	0.93	0.62	0.36	0.25	0.94	0.60	0.35	0.24	2.35	1.56	0.81	0.47	0.79	0.52	0.30	0.21
0.0001	0.0001	10.00	1.24	0.89	0.55	0.38	1.22	0.85	0.53	0.38	2.38	1.61	0.86	0.52	0.97	0.68	0.43	0.30
0.0001	0.0001	20.00	1.75	1.31	0.86	0.61	1.70	1.27	0.85	0.61	2.45	1.70	1.01	0.68	1.31	0.99	0.69	0.50
0.0001	-0.0001	1.00	0.82	0.50	0.28	0.19	0.83	0.51	0.28	0.19	3.11	2.05	1.06	0.60	0.80	0.49	0.27	0.18
0.0001	-0.0001	5.00	1.19	0.80	0.48	0.33	1.17	0.76	0.46	0.32	3.13	2.07	1.09	0.64	0.94	0.63	0.38	0.27
0.0001	-0.0001	10.00	1.67	1.21	0.76	0.53	1.64	1.15	0.74	0.52	3.16	2.11	1.17	0.72	1.22	0.89	0.58	0.42
0.0001	-0.0001	20.00	2.36	1.79	1.19	0.84	2.34	1.74	1.18	0.84	3.23	2.22	1.36	0.93	1.77	1.38	0.99	0.72
0.00001	0.00001	1.00	7.55	4.65	2.55	1.72	7.70	4.70	2.58	1.74	27.52	18.05	9.21	5.22	7.43	4.53	2.47	1.67
0.00001	0.00001	5.00	10.44	7.00	4.16	2.86	10.46	6.79	3.97	2.78	27.49	18.24	9.47	5.52	8.52	5.67	3.38	2.34
0.00001	0.00001	10.00	14.34	10.38	6.48	4.51	14.09	9.90	6.28	4.45	27.85	18.73	10.20	6.22	10.84	7.70	4.97	3.56
0.00001	0.00001	20.00	20.34	15.48	10.23	7.23	20.11	15.02	10.10	7.27	28.40	19.82	11.97	8.08	15.27	11.69	8.31	6.05
0.00001	-0.00001	1.00	7.65	4.71	2.60	1.75	7.83	4.78	2.61	1.75	28.07	18.61	9.54	5.35	7.48	4.60	2.52	1.70
0.00001	-0.00001	5.00	10.72	7.20	4.28	2.93	10.67	6.95	4.06	2.85	28.30	18.73	9.79	5.69	8.70	5.77	3.46	2.41
0.00001	-0.00001	10.00	14.86	10.66	6.70	4.67	14.52	10.19	6.47	4.59	28.47	19.16	10.53	6.45	11.04	7.93	5.15	3.67
0.00001	-0.00001	20.00	20.92	15.82	10.53	7.47	20.53	15.60	10.40	7.52	29.24	20.27	12.31	8.26	15.63	12.06	8.62	6.27

References

G. Abbate, U. Bernini, E. Ragozzino, and F. Somma. The temperature dependence of the refractive index of water. *Journal of Physics D: Applied Physics*, 11(8):1167–1172, 1978.

J. D. Gibbons and S. Chakraborti. *Nonparametric Statistical Inference*. CRC Press, Boca Raton, FL, fifth edition, 2011.

C.-Y. Tan and Y.-X. Huang. Dependence of refractive index on concentration and temperature in electrolyte solution, polar solution, nonpolar solution, and protein solution. *Journal of Chemical & Engineering Data*, 60(10):2827–2833, 2015.
